# Disentangling the Role of Deviant Letter Position on Cognate Word Processing

**DOI:** 10.3389/fpsyg.2021.731312

**Published:** 2021-09-24

**Authors:** Montserrat Comesaña, Juan Haro, Pedro Macizo, Pilar Ferré

**Affiliations:** ^1^Research Unit in Human Cognition, CIPsi, School of Psychology, University of Minho, Braga, Portugal; ^2^Center for Cognitive Science (C3), Nebrija University, Madrid, Spain; ^3^Department of Psychology and CRAMC, Universitat Rovira I Virgili, Tarragona, Spain; ^4^Department of Experimental Psychology and Mind, Brain and Behavior Research Center (CIMCYC), University of Granada, Granada, Spain

**Keywords:** letter-position encoding, cognate recognition, multilink, masked priming lexical decision task, 2-alternative forced-choice task

## Abstract

The way of coding letter position has been extensively assessed during the recognition of native words, leading to the development of a new generation of models that assume more flexible letter position coding schemes compared to classical computational models such as the interactive activation (IA) model. However, determining whether similar letter position encoding mechanisms occur during the bilingual word recognition has been largely less explored despite its implications for the leading model of bilingual word recognition (multilink) as it assumes the input-coding scheme of the IA model. In this study, we aimed to examine this issue through the manipulation of the position of the deviant letter of cognate words (external and internal letters). Two experiments were conducted with Catalan–Spanish bilinguals (a masked priming lexical decision task and a two-alternative forced-choice task) and their respective monolingual controls. The results revealed a differential processing for the first letter in comparison to the other letters as well as modulations as a function of language cue, suggesting amendments to the input-coding scheme of the multilink model.

## Introduction

In the last few decades, the way of coding letter position during visual native (L1) word recognition has been intensively examined (see, for instance, Chambers, 1979; Andrews, 1996; Perea et al., 2005; Gómez et al., 2008; see also Davis and Lupker, 2017 for an overview). The results of the studies on experimental effects such as letter transposition (e.g., judge-jugde), letter migration (e.g., beard-bread), letter substitution (e.g., face-fame), subset/superset (e.g., faulty-faculty), and backward priming (e.g., ecaf-face) uncovered that not all the letter positions in a word are equally processed. Thus, and contrary to the postulates of classical computational models (interactive activation [IA] model, McClelland and Rumelhart, 1981; Rumelhart and McClelland, 1982; dual-route cascaded [DRC] model, Coltheart et al., 2001; multiple readout model [MROM], Grainger and Jacobs, 1996), which assumed location-specific letter coding (i.e., they do not assign a special role to any letter position, and hence the positions are perfectly encoded), letters occupying middle and external positions within the word seem to be preferentially computed, showing a W-shaped function (e.g., Tydgat and Grainger, 2009; Ziegler et al., 2010)^1^. This phenomenon was explained by factors such as visual acuity (decreases from the fixation point toward more peripheral locations, see Anstis, 1998), and crowding effects (letters that occupy external positions in the string are processed more efficiently because they are flanked by only one letter). Thus, for instance, using a variety of three-field techniques (Humphreys et al., 1988) in which a visible-related or unrelated prime (in lowercase letters) was followed by a briefly (67 ms) presented uppercase word, Perea (1998) found larger priming effects (i.e., the difference between unrelated and related primes) for pairs of words whose deviant letter occupied an internal position (e.g., state-stale) than for pairs of words whose deviant letter was at the external (e.g., reach-react).

Overall, the findings collected on letter position coding during L1 word recognition led to the development of a new generation of models of visual word recognition, which employ more flexible orthographic input-coding schemes than those of classical models (e.g., LTRS model, Adelman, 2011; noisy Bayesian reader model, Norris et al., 2010; overlap model, Gómez et al., 2008; spatial coding model, Davis, 2010). For instance, according to the overlap model (Gómez et al., 2008), the identity of letters follows a normal distribution over the position. This way, the letter “D” in JUDGE is associated with position three but also, to a lesser degree, with positions two and three. As a result, compared to replacement-letter non-words (e.g., JUPTE AND JUDGE), transposed-letter non-words are more confusable with their base words (e.g., JUGDE and JUDGE) as it was empirically demonstrated in several languages and using different tasks and procedures (Perea et al., 2011; see Comesaña et al., 2016, for an overview; Yang et al., 2021; for differences between Indo-European and Semitic and Sino-Tibetan languages). Interestingly, this uncertainty about the position is reduced over time, therefore, distributions over positions only occur during the initial encoding process (i.e., when letter strings are presented very briefly and masked). Gómez et al. (2008) used a two-alternative forced-choice perceptual identification task−2AFCT (Ratcliff et al., 1989)—with manipulations of letter transpositions and letter replacements to examine the fit of the model to data. The lexical status of the stimuli presented (words vs. non-words) was also manipulated. In this task, participants are presented with a brief stimulus letter string (a cue), for ~50 ms, followed by a mask and then by two test letter strings (the cue and the foil), and have to decide which of the two test strings was presented earlier. The authors found a greater accuracy for the manipulations in which the first letter was altered than for the manipulations involving internal letters (e.g., sail-rail and slat-scat, respectively) regardless of the lexical status of stimuli (see Ratcliff, 1981, for the similar results) although the overall performance was better for target words than for non-target words. However, they failed to observe a preferential processing for the last letter over the internal ones (i.e., the accuracy for items varying in the last letter was similar to that for items varying in an internal letter). The authors stated that the absence of such preferential processing might be caused due to cue duration as this advantage was shown in previous studies using a cue duration >60 ms (the cue duration adopted by Gómez et al., 2008). The findings were taken as evidence of the importance of first letters for word recognition as Rayner and Pollatsek held several decades ago (Rayner and Pollatsek, 1989; Rayner et al., 2006; see also Tydgat and Grainger, 2009, for empirical support to the hypothesis of visual field specificity of receptive fields responsible for the first-position advantage). Nonetheless, the overlap model fitted the data pretty well (the fitting parameters for all the experiments conducted by the authors can be found in Gómez et al., 2008, p. 9, 46).

Another family of visual word recognition models makes similar predictions by assuming that there is a layer of “open bigrams” between the letter and word levels (open-bigram model, Grainger and van Heuven, 2004; multiple-route model, Grainger et al., 2012; and SERIOL model, Whitney, 2001). According to these models, transposed-letter words are more confusable than replacement-letter words because they share more open bigrams, which make them more similar at a perceptual level (e.g., JUDGE and JUGDE share more open bigrams [all except DG and GD] that JUDGE AND JUPTE). These models have more troubles, however, in accounting for backward priming effects (ecaf-face) observed in Sino-Tibetan languages (for more details, see Yang et al., 2021).

Although the way of coding letter position has been extensively examined in the literature on L1 word recognition, it has not been fully assessed in non-native or second language (L2) reading despite being a key issue for the front end of the leading model of bilingual visual word recognition: The multilink model (Dijkstra et al., 2019). Indeed, although multilink is a relevant model characterized by an integrated lexicon with a non-selective lexical access, it cannot account for the aforementioned effects (e.g., transposed-letter effects and letter substitution) as it incorporates, for the sake of simplicity, the same letter codification scheme as that of the IA model (i.e., a “position-specific” coding scheme). That is, it assumes that the positions of the letters are established very early in processing, and hence no letter position has a special role over the others. According to the model, the bottom-up activation of bilingual lexical representations is mainly determined by their overlap with the input. The aim of the present study was to test the front end of the multilink model by manipulating the deviant letter position of Spanish–Catalan cognate words (i.e., the translation equivalents that share the form besides the meaning). It is worth noting here that, although Catalan and Spanish are both alphabetic languages, the former has a deeper orthography than the latter. Such differences could impact letter position coding, an issue that we wanted to assess here. This is because cross-linguistic influences during the early stages of visual word recognition, especially from L1 to L2, are very well-documented (see, for instance, Sebastián-Gallés et al., 2006; Comesaña et al., 2012, 2015; Timmer and Schiller, 2012; Chen et al., 2020; Yang et al., 2021, for evidence of L1 influences on the orthographic coding system during L2 reading). In any case, if the multilink model is right, Spanish–Catalan cognate words like c*ifra*-x*ifra* (number) and *dan**z**a*-*dan**s**a* (dance), which differ in the position of the deviant letter, would be processed in the same way because both pairs differ in just one letter while maintaining the same degree of orthographic overlap (an normalized Levenshtein distance (NLD) of 0.80 for both word pairs). However, this seems not to be the case attending either to the results obtained from the monolingual domain (e.g., Mason, 1982; Tydgat and Grainger, 2009; Perea, 2015) or to the results obtained from a few bilingual studies developed so far in this matter (Font, 2001; Velan and Frost, 2007; Witzel et al., 2011; Lin and Lin, 2016; Chen et al., 2020; Yang et al., 2021; see, however, Comesaña et al., 2018, for null effects of the letter position on L2 cognate word recognition).

In a recent study, Lin and Lin (2016) focused on the processing of transposed-letter non-words to examine if transposition effects could be observed in native and non-native languages regardless of cross-language script. Chinese-English and Spanish-English bilinguals performed a mouse tracking trace task in which they had to decide whether a displayed item presented for 500 ms was a word or non-word by clicking with the mouse the “YES” or “NO” button. The results revealed transposed-letter effects in both L1 and L2 (i.e., participants took longer time to reject transposed-letter non-words as real words [lihgt, created from light] compared to replacement-letter non-words [lijst from light]). This effect was shown by the mouse trajectories as transposed-letter non-words were more strongly pulled toward the unselected alternative response option “YES” compared to replacement-letter non-words, revealing a lexical attraction to their base word and thus a greater processing speed cost. The magnitude of the transposed-letter effect was, however, modulated by the neighborhood size (items with fewer neighbors produced a larger effect than items with more neighbors; see Forster et al., 1987; Perea and Rosa, 2000; Kinoshita et al., 2009, for similar results in the monolingual domain), and by differences in script (the effect was higher for cross-script languages, probably because the position coding component of the orthographic coding system in Chinese is much more flexible than that of alphabetic languages; see Yang et al., 2021). Although the results of this study about letter position coding were interesting, they were found in case of non-words. As the processing of words and non-words follows different pathways (see Coltheart et al., 2001; see also Carreiras and Perea, 2002; Davis and Lupker, 2006), the question that remains unclear is whether or not the mechanism used by bilinguals to code letter position information in L2 words is essentially the same as that used by monolinguals in L1 words. One may think *a priori* that there is no reason to anticipate the differences in the orthographic processing of alphabetic languages in L2 or L1. However, the results obtained from the scarce research on this matter are inconclusive.

To the best of our knowledge, there are only two studies so far on letter position coding with bilinguals who used L2 words instead of non-words (Font, 2001; Comesaña et al., 2018). Both used the same task (lexical decision task) with bilinguals who speak languages with similar scripts, but obtained inconsistent results. Font (2001) examined letter position coding in French-Spanish bilinguals who were asked to decide whether or not a string of letters was a real Spanish (L2) word. Target words were French-Spanish cognates and their controls (non-cognate words—translation equivalents without form overlap like *maison*-casa [house]). The author observed that participants were faster to recognize cognate words in which the deviant letter position was at the end (e.g., texte-texto, the French and the Spanish words for *text*) than when it was within the word (e.g., usuel-usual, *usual*). Interestingly, the facilitation effect observed for cognates when compared to non-cognates was modulated by word frequency. Thus, when cognates had a low frequency in both languages, the facilitation effect for cognates whose deviant letter was in the middle of the words disappeared and tended toward inhibition.

In combination of a subsequent and highly controlled lexical decision study with the masked priming technique (English targets were preceded by the masked 50 ms related or unrelated European Portuguese [EP] primes; e.g., *coala*-KOALA vs. *passe* [pass]-KOALA), Comesaña et al. (2018) found no modulations in the size of the masked priming effect between EP cognate words that differ at the beginning vs. at the end of the word (e.g., *c**oala*-koala and *pape**l*-paper, respectively). The authors stated that, although the results were, *a priori*, consistent with the postulates of the multilink model, more research considering other letter positions and task requirements was needed. Indeed, the absence of differences between both groups of cognates could have been either due to the preferential processing of external letters already observed in the monolingual domain (e.g., Tydgat and Grainger, 2009) or to the feedback activation from a semantic to word form (note that both types of words share the meaning besides the form, and hence the feedback activation from the meaning to the form could be explained by the absence of differences between conditions). Another third and simpler possibility has to do with the fact that the masked priming effects are usually difficult to obtain under such fine-grained manipulations, as pointed out by Gómez et al. (2008, p. 21), especially when prime-target lexical frequency is matched as it was the case in the study of Comesaña et al., 2018 (see Perea, 1998 for evidence of modulations in the size of priming effects as a function of deviant letter position when the frequency of primes was higher than that of targets). These three hypotheses were examined in the carried out experiments.

In total, the main aim of the present research was to test the postulates of the front end of the multilink model regarding the way of coding the letter position during cognate word recognition by manipulating the position of the deviant letter (external and internal letters) of cognate words while maintaining constant their degree of orthographic overlap as well as the other variables that affect cognate processing. For that purpose, we carried out two studies with Catalan–Spanish bilinguals (Experiments 1a and 2a) and their respective monolingual controls (Experiments 1b and 2b) by using the most commonly employed tasks in the study of letter position coding during L1 and L2 word recognition [i.e., the masked priming lexical decision task (Experiment 1), and the 2AFCT (Experiment 2)]. The use of two different tasks allowed us to examine if the effects were modulated by task requirements.

In both tasks, five experimental conditions were created according to the location of the deviant letter: (a) initial (*xifra*-CIFRA [number]); second (*llebre*-LIEBRE [hare]); middle (*ploma*-PLUMA [feather]); penultimate (*dansa*-DANZA [dance]); and last (*rostre*-ROSTRO [face]). The predictions of the experiments were straightforward. We anticipated that the tenets of the multilink model regarding the encoding of letter position were not correct, and hence the differences between cognates as a function of deviant letter position in both tasks were expected. More precisely, if L2 word processing varies as a function of letter position (an unequivocal index of flexibility during letter position coding), a differential processing for cognates whose deviant letter occupy the first position within the word in comparison with cognates whose deviant letter in any other position would be observed at least in the more perceptual task (i.e., the 2AFCT).

## Experiment 1A

The aim of the first experiment was to replicate the study of Comesaña et al. (2018) with Catalan–Spanish cognate words that vary in external letters (e.g., xifra-cifra and rostre-rostro), and to extend it to cognate words whose deviant letter is within the word (e.g., llebre-LIEBRE, ploma-PLUMA, and dansa-DANZA). If letter position during L2 word recognition matters and the masked priming procedure with a brief prime duration (50 ms) is robust enough to capture the manipulations of letter position, we expected to find greater priming effects for cognates whose deviant letter is within the word as it was observed using L1 neighbor words through the use of a three-field technique with unmasked primes (see Perea, 1998).

### Method

#### Participants

About 40 undergraduate students (34 women and 6 men, mean age 22.3 years, SD = 6.4) from the Universitat Rovira i Virgili (Tarragona, Spain) participated in the experiment in exchange for academic credits (all of them signed an informed consent). The students were highly proficient Catalan–Spanish bilinguals and had Catalan as their dominant and preferred language. To assess their proficiency in both languages, they were asked to complete a questionnaire in which they had to rate their ability in listening, speaking, reading, and writing by using a seven-point Likert scale (1 = “very poor,” 7 = “native”; see Table 1).

**Table 1:** Mean level of proficiency in the four linguistic skills in Spanish (standard deviation in parentheses) of the participants of Experiment 1a and Experiment 1b.

	**Experiment 1a**		**Experiment 1b**
	**Catalan**	**Spanish**	**Spanish**
**Skills**	**Mean** **(***SD***)**	**Mean** **(***SD***)**	**Mean** **(***SD***)**
Listening	6.97 (0.16)	6.95 (0.22)	6.59 (0.67)
Speaking	6.79 (0.61)	6.33 (0.90)	6.38 (0.71)
Reading	6.90 (0.38)	6.79 (0.47)	6.53 (0.62)
Writing	6.74 (0.68)	6.59 (0.64)	6.41 (0.61)
Average	6.85 (0.39)	6.67 (0.43)	6.48 (0.54)

*The anchor points for the 7-point Likert scale were 1 = “very poor,” and 7 = “native.”*

To evaluate their language dominance, they were asked to indicate which of the two languages was preferred and was used more frequently in different contexts (listening, speaking, reading, and writing). To make their ratings, they were provided with another seven-point Likert scale, where one was “Only in Catalan” and seven was “Only in Spanish.” The mean ratings of preference and frequency showed that Catalan was the dominant language of the participants: preference (*M* = 3.24, SD = 0.73, range = 1.75–5) and frequency (*M* = 2.96, SD = 0.70, range = 2–4). It is worth noting here that four is the middle point of the scale, which indicates a total equality in the preference and usage of both languages.

#### Design and Materials

Critical stimuli consisted of 240 Catalan prime-Spanish target translation pairs. Half of these pairs were Catalan–Spanish cognate translations (e.g., *correu-correo* [mail], respectively), and the other half were non-cognate translations (e.g., *blat-trigo* [wheat]). Cognate translation pairs were divided into five experimental conditions according to the position of the letter in which the translation equivalents had a difference (hereafter, *deviant letter position*): initial position (e.g., *xifra-cifra* [number]), second position (e.g., *llebre-liebre* [hare]), middle position (e.g., *ploma-pluma* [feather]), penultimate position (e.g., *dansa-danza* [dance]), and last position (e.g., *rostre-rostro* [face]). Targets from cognate and non-cognate conditions, as well as among cognate conditions, were matched in frequency, word length, and the number of orthographic neighbors (all *ps* > 0.42; see Table 2 for stimuli characteristics). These values were taken from the EsPal database (Duchon et al., 2013).

**Table 2:** Characteristics of the stimuli used in the experiment (standard deviations are shown in parentheses).

	**Log frequency (0.03–2.84)**			**Word length (2–10)**			**Neighbors (0–29)**			**NLD (0.00–0.89)**
	**Trans**.		**Unrel**.	**Trans**.		**Unrel**.	**Trans**.		**Unrel**.	
**Condition**	**T**	**P**	**P**	**T**	**P**	**P**	**T**	**P**	**P**	
Cognates, initial	1.04	1.07	1.06	6.5	6.5	6.5	4.71	7.17	6.33	0.84
	(0.43)	(0.41)	(0.41)	(1.25)	(1.25)	(1.25)	(3.28)	(4.58)	(5.16)	(0.03)
Cognates, second	1.19	1.18	1.13	6.25	6.25	6.63	5.17	9.67	9.92	0.83
	(0.63)	(0.58)	(0.39)	(1.36)	(1.36)	(1.44)	(4.30)	(6.62)	(6.95)	(0.03)
Cognates, middle	1.14	1.16	1.12	6.38	6.38	6.33	5.5	8.92	8.04	0.83
	(0.43)	(0.45)	(0.60)	(1.41)	(1.41)	(1.37)	(4.87)	(5.28)	(6.55)	(0.04)
Cognates, penultimate	1.04	1.14	1.08	6.63	6.63	6.42	4.46	7.96	7.46	0.84
	(0.56)	(0.42)	(0.41)	(1.44)	(1.44)	(1.38)	(3.16)	(4.91)	(6.19)	(0.04)
Cognates, last	1.26	1.2	1.16	6.08	6.08	6.08	5.54	7.5	7.38	0.83
	(0.59)	(0.41)	(0.41)	(1.35)	(1.35)	(1.35)	(6.39)	(4.60)	(6.48)	(0.04)
Non-cognates	1.08	1.15	1.14	6.52	6.31	6.32	4.75	9.04	9.83	0.2
	(0.50)	(0.54)	(0.54)	(1.50)	(1.81)	(1.82)	(3.84)	(9.68)	(10.02)	(0.14)

*For cognate conditions, the position of the deviant letter is indicated after the comma. The range of values per variable is indicated below the variable name*.

*Trans., Translation condition; Unrel, Unrelated condition; T, Target; P, Prime*.

Likewise, primes from cognate and non-cognate conditions, as well as among cognate conditions, were equated in frequency, word length, and the number of orthographic neighbors (all *ps* > 0.43). However, given that primes were Catalan words, we obtained their values from a different source of that of targets (i.e., NIM database, Guasch et al., 2013). Both the frequencies of Spanish target words (*M* = 1.11) and Catalan prime words (*M* = 1.15) were based on the logarithm of the frequency per million words and did not differ significantly from each other, *t* < 1.8. In addition, the orthographic overlap between cognate targets and their primes, measured as NLD (Levenshtein, 1966; Schepens et al., 2012), was the same among cognate conditions, *F*_(4, 119)_ = 0.64, *p* = 0.64, but different between cognate and non-cognate conditions (0.84 and 0.20, respectively, *t* = 47.94). Finally, given that orthotactical markers (the sublexical properties of words, which are specific to one of the two languages of an bilingual) reduce cross-linguistic transfer (Sebastián-Gallés et al., 2006; Casaponsa et al., 2019), in this study, this factor was controlled as much as possible across conditions.

On the other hand, we selected 240 Catalan words to create the unrelated prime conditions. Each of these words was of the same length and approximately the same frequency as the Catalan prime it replaced (e.g., *canal* [channel], and was selected as an unrelated prime for the pair *ploma-pluma* [feather]). Hence, the primes for translation and unrelated conditions were equivalent in log frequency and word length (all *ps* > 0.59). Finally, a set of 240 orthographically non-legal words were created by replacing one letter from cognate and non-cognate Spanish words (e.g., the non-word *birro* was created from the non-cognate word *barro* [mud], whereas the non-word *tero* was created from the cognate word *cero*, “zero”). Word length was matched as much as possible between non-word targets (*M* = 6.7) and word targets (*M* = 6.44), *t* < 1.7. Each non-word target was preceded by a Catalan word prime. Half of these primes were the Catalan translation of the Spanish word that was used to create the non-word (e.g., *fang*, which is the translation of the Spanish word *barro*, served as prime for the non-word *birro*). The other half were unrelated primes of the same length and frequency like the related primes. This was done to maintain a similar orthographic overlap between primes and targets in non-word conditions as that used in word conditions. Finally, we constructed two counterbalanced lists of stimuli so that the 240 target words appeared under the two priming conditions (translation or unrelated) across participants, but the participants did not see any prime or target more than once. That is, if a target was presented with its translation prime on the first list, it was presented with its unrelated prime on the second list and *vice versa*.

#### Procedure

The experiment was run using the DMDX software (Forster and Forster, 2003). All participants completed a lexical decision task. Each trial consisted of the following steps. Firstly, a forward mask (e.g., “##########”) with the same length as the longest word of the prime-target pair was presented (i.e., 10 characters). The mask remained on the center of the screen for 500 ms, and was then replaced by the prime stimulus. The prime was presented for 50 ms in lowercase and was replaced with an uppercase target, which was a string of letters representing either a word or non-word. At that point, participants had to decide whether the target was a Spanish word or not by pressing one of the two buttons of a keypad as a fast and possible attempt not to commit errors. The string of letters remained on the screen until the response of participants or a timeout of 2,500 ms. After that, a new trial was displayed to be preceded by a 1,000 ms interval. Each participant was presented with a different random order of stimuli. There were 480 experimental trials and 12 practice trials. Experimental trials were divided into four blocks. Between the blocks, participants were allowed to take a short break.

### Results and Discussion

We removed the data from the four participants with more than 15% of the errors (two participants in each list). Thus, the final sample was 36 participants. In addition, reaction times that exceeded 2 SD of the mean of each participant and those faster than 300 ms or slower than 2,000 ms were removed (6.3% of the whole). Then, we calculated the mean of response times (RTs) for the correct responses and the mean of error rates (%*E*) across experimental conditions (see Table 3). Both RTs and %*E* were analyzed using ANOVAs^2^. Alpha was set to 0.05 for all analyses, and multiple comparisons were Bonferroni corrected. Two analyses were carried out: The first one examined the cognate status effect (i.e., if there were differences between cognates and non-cognates as well as if masked priming effects were greater for the former ones, as typically observed in the literature). The second one, restricted to cognate words, examined the critical question at stake (i.e., if the priming effect size was modulated by the position of the deviant letter).

**Table 3:** Mean response times (RTs; in milliseconds) and percentage of errors (% E) in the different experimental conditions of Experiment 1a.

	**Translation**		**Unrelated**		**Priming effects**	
	**RT**	**%E**	**RT**	**%E**	**RT**	**%E**
Cognates, initial	765.90 (19.65)	8.23 (1.8)	811.31 (20.46)	9.92 (1.5)	45.41	1.69
Cognates, second	736.10 (18.46)	4.34 (0.94)	770.90 (17.41)	6.190 (1.54)	34.80	1.85
Cognates, middle	733.96 (15.35)	5.12 (1.01)	788.15 (17.03)	12.52 (1.84)	54.19	7.40
Cognates, penultimate	708.87 (17.83)	4.05 (1.26)	772.11 (15.01)	4.06 (1.09)	63.24	0.01
Cognates, last	735.86 (16.43)	9.37 (2.21)	769.26 (16.34)	11.55 (2.09)	33.40	2.18
Non-cognates	753.27 (14.12)	4.71 (0.64)	767.26 (15.91)	5.23 (0.91)	13.99	0.52

*Standard errors are presented in parentheses*.

#### Cognate Status

In the first ANOVA, target words were analyzed using a cognate status (cognate or non-cognate) × prime relatedness (translation or unrelated) × list of stimuli (list 1 or 2) design. In the analysis by participants, cognate status and prime relatedness were a within-group factor, and list of stimuli was included as a between-group factor. In the analysis by items, prime relatedness and list of stimuli were included as a within-group factor, whereas cognate status was a between-group factor. Only the analyses that were significant by subjects and items are reported.

ANOVA showed a main effect of prime relatedness as translation primes (mean RTs = 745 ms; mean %*E* = 5.45%) facilitated word recognition in comparison to unrelated primes (mean RTs = 775 ms, mean %*E* = 7.04%) in latency data, *F*_1(1, 35)_ = 42.42, MSE = 32,620, *p* < 0.001, $\eta_{\text{p}}^{2}$ = 0.55, *F*_2(1, 238)_ = 46.45, MSE = 122,438, *p* < 0.001, *η*_*p*_^2^ = 0.16, and in error data, *F*_1(1, 35)_ = 8.88, MSE = 89.02, *p* = 0.005, *η*_*p*_^2^ = 0.20, *F*_2(1, 238)_ = 7.90, MSE = 300.68, *p* = 0.005, *η*_*p*_^2^ = 0.03. In addition, a significant effect of cognate status appeared in error data, *F*_1(1, 35)_ = 30.2, MSE = 237.52, *p* < 0.001, *η*_*p*_^2^ = 0.46, *F*_2(1, 238)_ = 4.03, MSE = 757.27, *p* = 0.046, *η*_*p*_^2^ = 0.02, but not in latency data (both *p*s > 0.77, mean RTs for cognates = 759 ms, mean RTs for non-cognates = 760 ms), indicating that cognate words were recognized less accurately (*M* = 7.53%) compared to non-cognate words (*M* = 4.97%). This inhibitory effect for cognate over non-cognate words usually appear when identical cognates are not included in the experimental list, as it was the case here [see Comesaña et al. (2015) for an overview of list composition effects in cognate processing; also Comesaña et al. (2012) for converging electrophysiological evidence]. Furthermore, the analysis of the latency data revealed an interaction between prime relatedness and cognate status, *F*_1(1, 35)_ = 14.60, MSE = 9,349, *p* = 0.001, *η*_*p*_^2^ = 0.29, *F*_2(1, 238)_ = 16.93, MSE = 44,638, *p* < 0.001, *η*_*p*_^2^ = 0.07. Although there was a significant priming effect (i.e., the difference between unrelated and translation conditions) for both cognates and non-cognates (all *p*s < 0.05), the effect was significantly larger for the former in comparison to the latter, as expected (46.22 and 13.99 ms, respectively), *t*_1(35)_ = 3.82, *p* = 0.001, *t*_2(238)_ = 4.12, *p* < 0.001.

#### Deviant Letter Position

In the second ANOVA, only cognate target words were analyzed using a deviant letter position (initial, second, middle, penultimate, or last) × prime relatedness (translation or unrelated) × list of stimuli (lists 1 or 2) design. In the analysis by participants, deviant letter position and prime relatedness were treated as within-group factors, whereas list of stimuli was included as a between-group factor. In the analysis by items, prime relatedness and list of stimuli were included as within-group factors, and deviant letter position was treated as a between-group factor. The results revealed a main effect of prime relatedness in latency data as translation primes (mean RTs = 736 ms, mean %*E* = 6.22%) facilitated word recognition with respect to unrelated primes (mean RTs = 782 ms, mean %*E* = 8.85%), *F*_1(1, 35)_ = 58.15, MSE = 192,240, *p* < 0.001, *η*_*p*_^2^ = 0.62, *F*_2(1, 234)_ = 63.15, MSE = 166,741, *p* < 0.001, *η*_*p*_^2^ = 0.21, and in error data, *F*_1(1, 35)_ = 8.10, MSE = 619.11, *p* = 0.007, *η*_*p*_^2^ = 0.19, *F*_2(1, 234)_ = 11.56, MSE = 431.81, *p* = 0.001, *η*_*p*_^2^ = 0.05.

**Figure 1:**
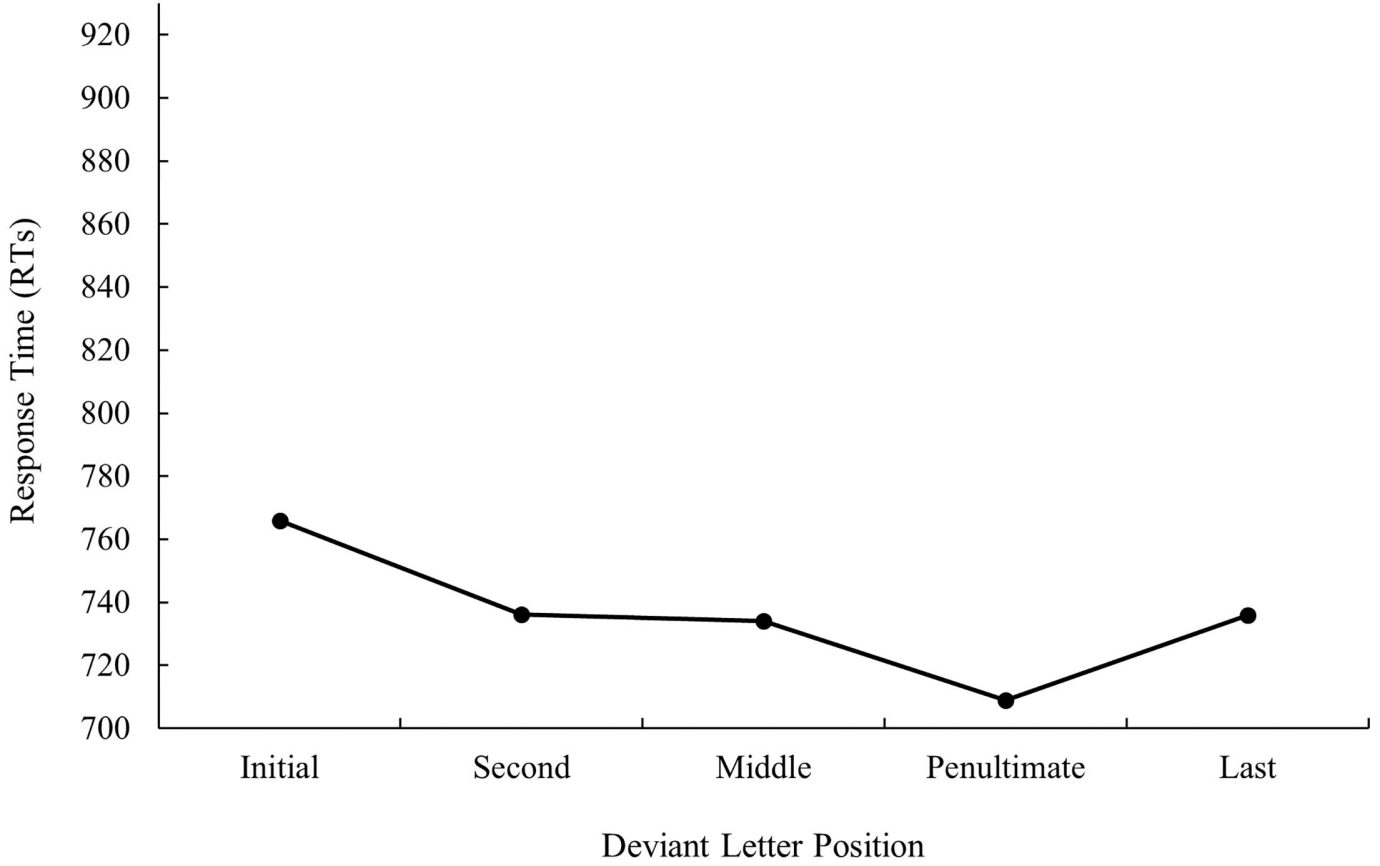
Mean response times (RTs; in ms) for cognate words in the related condition according to its deviant letter position in Experiment 1a.

**Figure 2:**
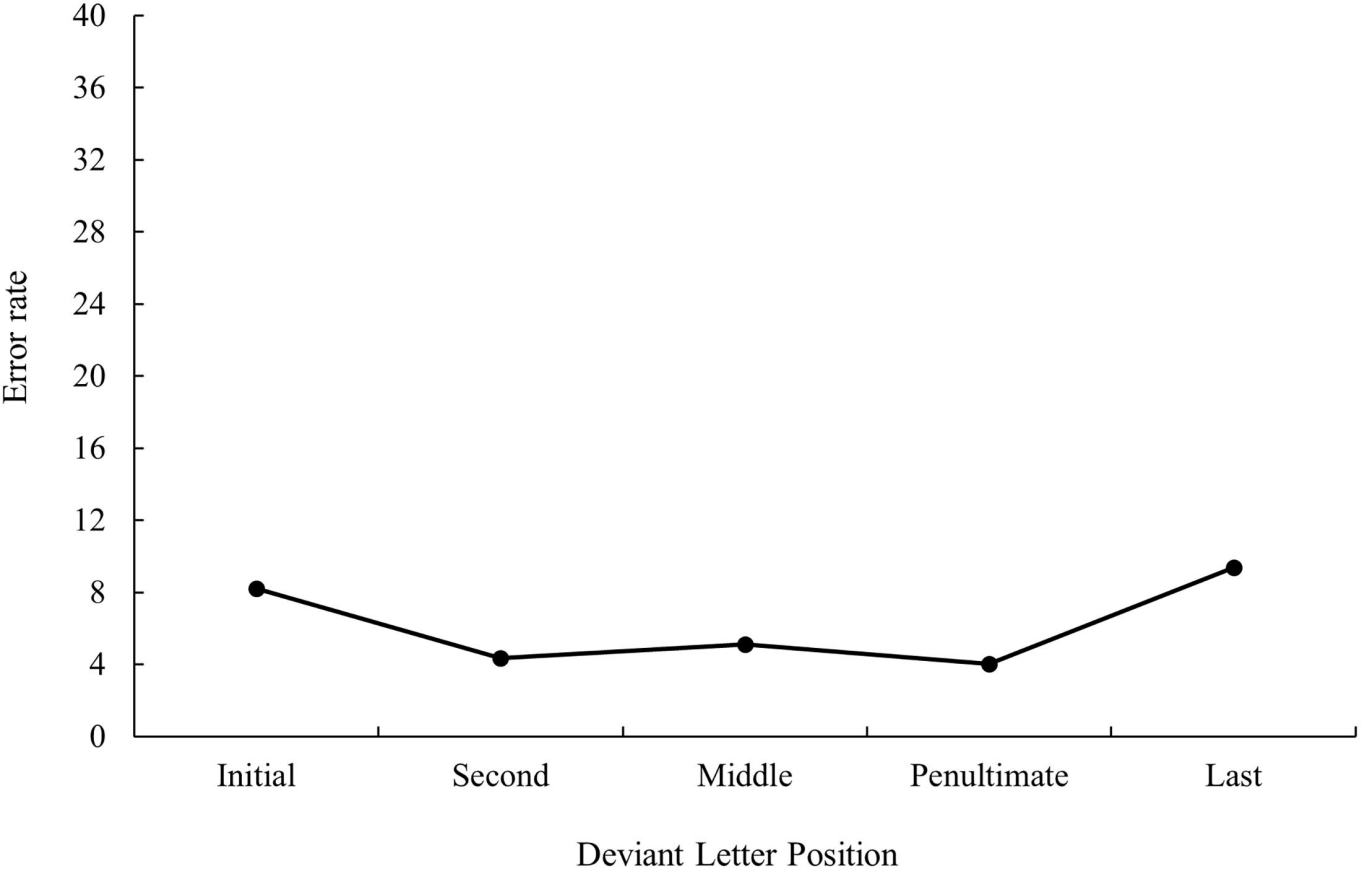
Mean error rate (in percentage) for cognate words in the related condition according to its deviant letter position in Experiment 1a.

The main effect of deviant letter position was significant in the analysis of error data, *F*_1(4, 140)_ = 11.97, MSE = 536.56, *p* < 0.001, *η*_*p*_^2^ = 0.26, *F*_2(5, 234)_ = 2.34, MSE = 433.11, *p* = 0.043, *η*_*p*_^2^ = 0.05. Cognate words with the deviant letter in the first and last position had a higher percentage of errors (9.08 and 10.46%, respectively) in comparison with cognates whose deviant letter position was within the word (second and penultimate: 5.27 and 4.06%, respectively, all *p*s < 0.05). No significant differences were found between cognate words with the deviant letter in the first and last position as well as between cognate words with the deviant letter in the second and penultimate position (all *p*s > 0.05). In addition, cognates with the deviant letter in the penultimate place were more accurately recognized than cognates whose deviant letter was in the middle (4.06 and 8.82%, respectively, *p* < 0.05). The graphical representation of deviant letter position in the translation condition on RTs and % of errors is shown in Figures 1, 2, respectively.

That is, when considering the main effect of deviant letter position, cognate words in which external and middle letters were the deviant, produced less precise responses in comparison to internal letters regardless of prime type, showing the W-shaped function observed in the monolingual domain with a target search task (see, for instance, Mason, 1982) or the bare-probe identification procedure (i.e., when a target letter presented previously within a letter string has to be identified after the presentation of a bare-probe signaling the position of report; see Tydgat and Grainger, 2009). However, the interaction effect between deviant letter position and prime relatedness was not significant either in latency or in error data (all *p*s > 0.1), and hence no differences on the priming effect size regarding the position of the deviant letter were observed.

The null effect of deviant letter position on the magnitude of priming is consistent with the findings found in the study of Comesaña et al. (2018) with EP-English bilinguals. Note that we followed a similar procedure in the selection of materials as that followed by Comesaña et al. (2018) (i.e., cognate words from different conditions were carefully matched for a number of important sublexical and lexical variables that affect processing). Besides, although in the monolingual domain, there are some studies showing modulations in priming effects as a function of deviant letter position when the prime is visible (see Perea, 1998), these effects are usually small and difficult to obtain when the prime is brief (50 ms) and masked, as pointed out by Gómez et al. (2008, p. 21). The usage of a more perceptual task, such as the 2AFCT, may be therefore more informative. In this task, the differences in accuracy among experimental conditions are usually large and graded and thus easily measurable. Indeed, flexible input-coding schemes such as the overlap model were originally applied to data from perceptual tasks like the 2AFCT with the manipulations of letter replacements and letter transpositions (see Gómez et al., 2008). Therefore, the aim of the second experiment was to further examine letter position coding during cognate word recognition through the use of a 2AFCT.

However, before presenting the 2AFCT experiment and establishing firm conclusions, it is important to examine the contribution of meaning overlap in the results found in Experiment 1a (note that the words used were translation equivalents, and hence they shared the meaning besides the form). The overlap in meaning across cognate conditions may have attenuated the differences attributed to deviant letter position in the masked priming effect. Experiment 1b was precisely designed to explore this issue through the replication of Experiment 1a with a control group of native speakers of Spanish with no knowledge of Catalan. In this way, we canceled the influence of meaning overlap between prime and target pairs as the primes for monolinguals were non-words. We expected to find a similar pattern of results with cognate words as that observed with Catalan–Spanish bilinguals if meaning overlap was not affecting the findings. Additionally, this experiment allowed us to rule out the possible influence of artifacts in the materials. If they are well-constructed, priming effects would be restricted to cognate words due to the presence of form overlap between the prime and target, and would not be observed with non-cognates (see, for instance, Forster, 1987; also Perea and Lupker, 2003, for more details on masked form priming).

## Experiment 1B

### Method

#### Participants

About 32 native speakers of Spanish (26 women and 6 men, mean age 22.84 years, SD = 3.47) participated in the experiment in exchange for academic credits. They were undergraduate students from the University of Granada (Granada, Spain). Participants were asked to fill in a questionnaire similar to that of Experiment 1, in which they had to rate their ability in several languages (i.e., Spanish, English, French, and Catalan) in listening, speaking, reading, and writing by using a seven-point Likert scale (1 = “very poor” in the assessed skill, 7 = “native”). According to the ratings of the questionnaire, none of the participants had knowledge of Catalan. Fluency of participants in Spanish is reported in Table 1.

#### Materials and Procedure

The same set of materials and procedure as in Experiment 1a were used in this experiment.

### Results and Discussion

None of the participants were removed from the analyses based on their error rate (all participants made <15% of the errors). As mentioned in Experiment 1a, RTs that exceeded 2 SD of the mean of each participant and those faster than 300 ms or slower than 2,000 ms were removed (<6% of the whole). Then, we calculated the mean RTs for the correct responses and the mean %*E* across experimental conditions (see Table 4). We conducted the same analyses as in Experiment 1a.

**Table 4:** Mean response times (RTs; in milliseconds) and percentage of errors (% E) in the different experimental conditions of Experiment 1b.

	**Translation**		**Unrelated**		**Priming Effects**	
	**RT**	**%E**	**RT**	**%E**	**RT**	**%E**
Cognates, initial	783.72 (31.11)	6.39 (1.26)	812.34 (29.59)	6.13 (1.71)	28.62	−0.26
Cognates, second	756.69 (27.63)	2.34 (0.86)	775.22 (28.20)	3.14 (0.90)	18.53	0.80
Cognates, middle	741.29 (24.50)	6.01 (1.41)	772.86 (25.73)	6.94 (1.41)	31.57	0.93
Cognates, penultimate	723.47 (24.45)	1.09 (0.52)	780.25 (28.58)	2.98 (1.09)	56.78	1.89
Cognates, last	727.96 (24.87)	7.95 (2.26)	772.10 (25.01)	5.67 (1.47)	44.14	−2.28
Non-cognates	755.69 (24.05)	3.02 (0.49)	768.79 (25.72)	4.12 (0.66)	13.10	1.10

*Standard errors are presented in parentheses*.

#### Cognate Status

Latency data analyses showed a main effect of prime relatedness as targets preceded by related primes were recognized faster (751 ms) than targets preceded by control unrelated primes (776 ms), *F*_1(1, 31)_ = 30.82, MSE = 19,227, *p* < 0.001, *η*_*p*_^2^ = 0.50, *F*_2(1, 238)_ = 35.62, MSE = 79,242, *p* < 0.001, *η*_*p*_^2^ = 0.13. As expected, the main effect of cognate status was not significant either in RTs or in %*E* (all *p*s > 0.05). In addition, as suggested by the interaction between prime relatedness and cognate status on latency data, *F*_1(1, 31)_ = 6.56, MSE = 4,169, *p* = 0.02, *η*_*p*_^2^ = 0.18, *F*_2(1, 238)_ = 10.89, MSE = 24,233, *p* = 0.001, *η*_*p*_^2^ = 0.04, such relatedness effect was observed only for cognates (all *p*s < 0.05). No other significant main effects or interactions were found either for RTs or for %*E*.

#### Deviant Letter Position

**Figure 3:**
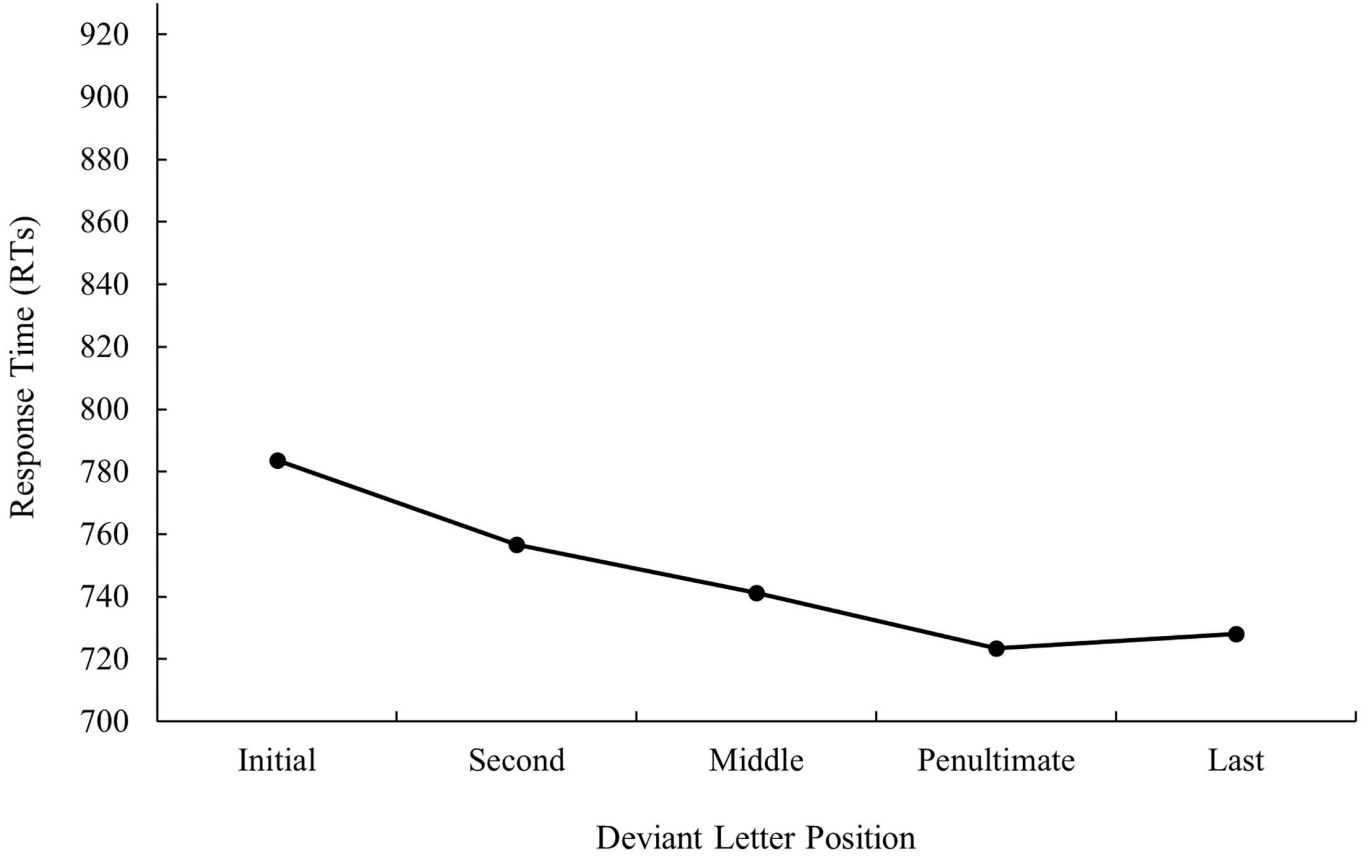
Mean response times (RTs; in ms) for cognate words in the translation condition according to its deviant letter position in Experiment 1b.

**Figure 4:**
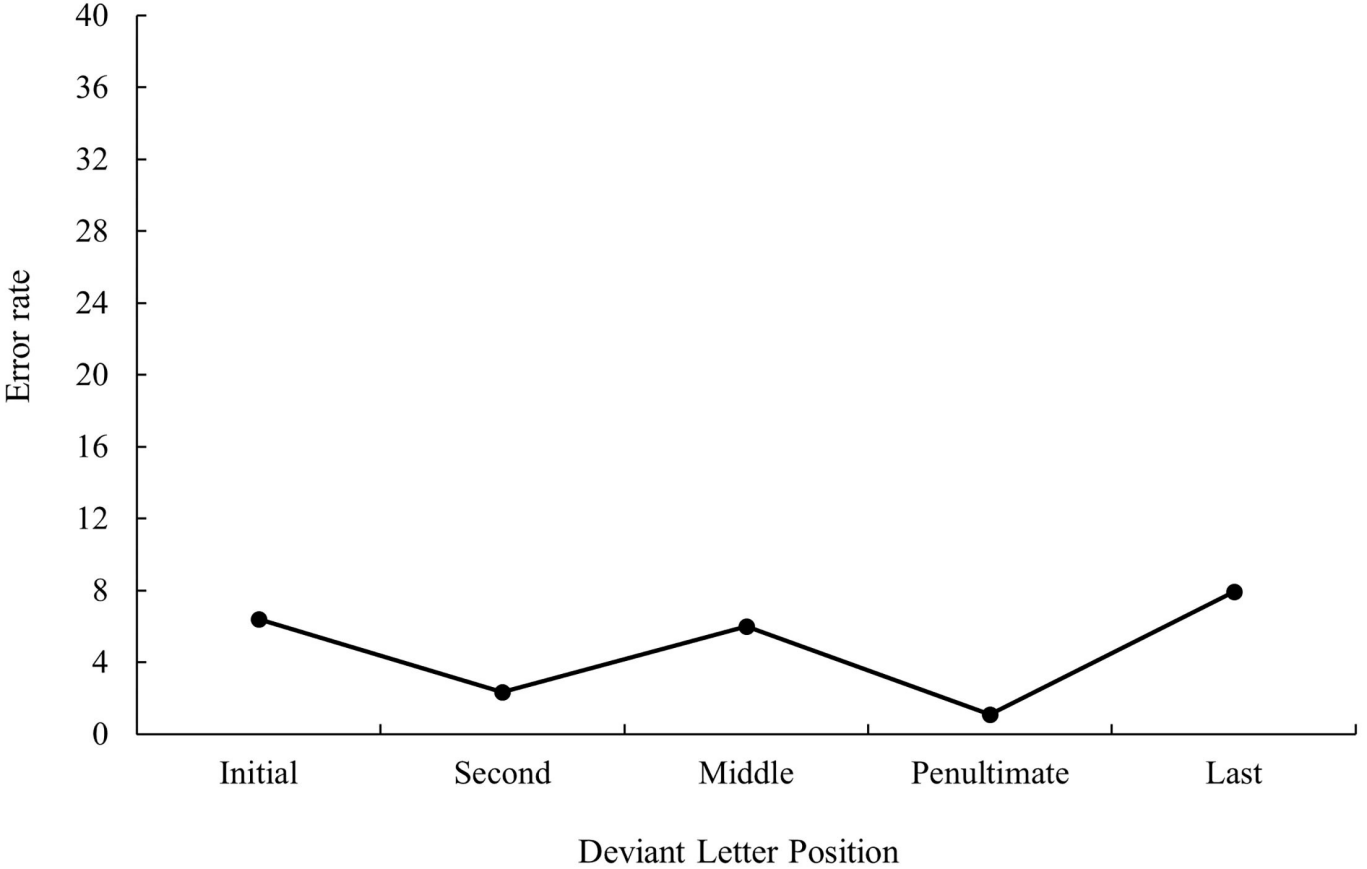
Mean error rate (in percentage) for cognate words in the related condition according to its deviant letter position in Experiment 1b.

ANOVA showed a main effect of prime relatedness in latency data as targets preceded by translation primes were recognized faster than targets preceded by unrelated primes (747 and 783 ms, respectively), *F*_1(1, 31)_ = 30.48, MSE = 103,260, *p* < 0.001, *η*_*p*_^2^ = 0.50, *F*_2(1, 115)_ = 38.57, MSE = 95,559, *p* < 0.001, *η*_*p*_^2^ = 0.25 (the effect was not significant in the analysis of error data; all *p*s > 0.05). Although the main effect of deviant letter position was not significant either in the analysis of latency data or in the analysis of error data, the pattern was very similar to that of bilingual participants from Experiment 1a, as can be seen in Figures 3, 4 (for latency and error data, respectively). Its non-significance may be due to the low number of subjects in comparison with those from Experiment 1a along with the high variability observed. Indeed, on analyzing the data for monolinguals and bilinguals within the same model, the effect disappears^3^.

As presented in Experiment 1a, there were no modulations in masked priming effects as a function of deviant letter position, either in the latency or in error data (all *p* > 0.05).

The results of this subexperiment were clear-cut: priming effects in the native speakers of Spanish who had no knowledge of Catalan were essential due to a form overlap between the prime and target (as no effect was observed for non-cognates) and were not modulated by deviant letter position. Indeed, the pattern of results was similar to that observed in Experiment 1a with bilinguals. This allowed us to rule out the existence of artifacts in the materials.

The most relevant result of Experiment 1 was the replication of the null effect of deviant letter position in masked priming (Comesaña et al., 2018). Although the absence of interaction between the two factors does not legitimate us to do planned comparisons across conditions, it is important to note that in both populations (monolinguals and bilinguals), the size of masked priming tended to be greater for cognates varying in the middle letter. In addition, the magnitude of the effect was very similar in bilinguals and monolinguals (55 and 56 ms, respectively). Besides, when considering the main effect of deviant letter position, both groups of participants showed a W-shaped function (although it was only significant for the bilingual group; see Figures 1–4). Overall, the cognates that differ in their first letter were slower and less precisely recognized than the other cognate conditions regardless of the prime type. This could be possible due to the existence of similar mechanisms underlying the way in which letter position is coded in L1 and L2. We recognize, however, that we should be cautious with this interpretation due to the absence of modulations in the size of priming across conditions, and hence a second experiment was needed. We decided to carry out a more perceptual task: the 2AFCT in which a cue word (e.g., *cerveza* [beer]) was briefly presented (50 ms) and followed by the same word (*cerve**z**a*) and its Catalan translation (*cerve*s*a*). Participants had to guess which of the two words was presented previously. We opted for this task not only because it seems to be more informative than the masked priming lexical decision task (note that the differences in accuracy among experimental conditions are usually large and graded and thus easily measurable; see Gómez et al., 2008), but also because, in this way, we could assess whether the results were modulated by task requirements using the same prime duration across tasks (50 ms).

## Experiment 2A

The aim of this second experiment was to further examine the way of coding letter position in bilingual word recognition by using a more perceptual task, i.e., a 2AFCT (participants had to guess between the two target alternatives, the one was previously presented for 50 ms). The predictions were as follows: if internal letters have a higher perceptual noise than external and middle ones and, as a consequence, a higher confusability, the pattern of results would be just the opposite as that observed using the masked priming lexical decision task (i.e., an inverted W-shaped function). This is because, whereas in the masked priming technique, the higher the similarity between the two strings (prime-target), the higher the activation of the target, and hence the faster the responses, in the 2AFC task the higher the similarity of the two alternatives, the slower the responses as it is difficult to distinguish between the two very similar alternatives. That is, cognates varying in internal letters exhibit slower responses and more errors than those varying in external and middle letters. Although, it is worth noting here that as we used a cue duration lower than 60 ms, and the differences between cognate conditions may reach significance only when considering the first-letter condition (see Gómez et al., 2008).

### Method

#### Participants

About 40 undergraduate students (39 women and 1 men, mean age 21.03 years, SD = 3.76) from the Universitat Rovira i Virgili (Tarragona, Spain) participated in the experiment in exchange for academic credits (all of them signed an informed consent). These participants were very similar to those who participated in Experiment 1, all of them being highly proficient Catalan–Spanish bilinguals. None of the participants in Experiment 2a took part in Experiment 1a. To assess their proficiency in both languages, as well as their frequency of use and preference for each language, participants were asked to complete the same questionnaire as that used in Experiment 1a (see Table 5). The mean questionnaire ratings of preference and frequency showed that Catalan was the dominant language of the participants: preference (*M* = 3.30, SD = 0.72, range = 1–4) and frequency (*M* = 3.42, SD = 0.63, range = 2–4.75). Bearing in mind that four is the middle point the seven-point Likert scale, which indicates a total equality in the preference and usage both languages (1 = “Only in Catalan” and 7 = “Only in Spanish”).

**Table 5:** Mean level of proficiency in the four linguistic skills in Catalan and Spanish (standard deviation in parentheses) of the participants of Experiment 2a and Experiment 2b.

	**Experiment 2a**		**Experiment 2b**
	**Catalan**	**Spanish**	**Spanish**
**Skills**	**Mean** **(***SD***)**	**Mean** **(***SD***)**	**Mean** **(***SD***)**
Listening	6.95 (0.22)	6.92 (0.32)	6.69 (0.59)
Speaking	6.92 (0.35)	6.92 (0.30)	6.34 (0.87)
Reading	6.92 (0.35)	6.91 (0.34)	6.63 (0.61)
Writing	6.85 (0.43)	6.92 (0.31)	6.22 (0.75)
Average	6.91 (0.34)	6.92 (0.32)	6.47 (0.70)

*The anchor points for the 7-point Likert scale were 1 = “very poor,” and 7 = “native.”*

#### Materials

The 240 Catalan–Spanish translation pairs similar to those in Experiments 1a and 1b were used in this experiment.

#### Procedure

The experiment was run using the DMDX software (Forster and Forster, 2003). Participants completed a 2AFC task similar to that used by Gómez et al. (2008). Each trial began with a fixation point (“+”) displayed at the center of the screen for 500 ms. After that, a word in uppercase letters (hereafter cue word) was presented for 50 ms. It was one of the two members of the critical pairs (i.e., either the Catalan word, *cervesa*, or the Spanish word, *cerveza* [beer]). The cue word was immediately masked with segments of letters. Then, the two words in lowercase letters appeared simultaneously below the mask, one to its right and another to its left. These words were the masked (cue) word and its translation (e.g., *cervesa-cerveza*). Participants had to decide which of the two words was presented before the mask (i.e., which was the cue word). They were instructed to press the right button of a keypad if the target word was the one that is located on the right, and to press the left button if it was the one that is located on the left. After response or timeout (3,000 ms), the next trial started automatically. We constructed four different versions of the experiment to counterbalance the language of the cue (i.e., Catalan or Spanish) and its position (i.e., left or right) across participants. Therefore, each participant saw each cue only once. There were 240 experimental trials and 12 practice trials. Experimental trials were divided into three blocks. Between blocks, participants could take a short break.

### Results and Discussion

The data from the three participants having more than 30% of the errors (each from a different version of the experiment) were discarded from analyses. In addition, RTs faster than 250 ms or slower than 1,600 ms were removed (<4% of the whole). Then, we calculated the mean RTs for the correct responses and the mean of %*E* across experimental conditions (see Table 6).

**Table 6:** Mean response times (RTs; in milliseconds) and percentage of errors (% E) in the different experimental conditions of Experiment 2a.

	**Catalan cue**		**Spanish cue**		**Total**	
	**RT**	**%E**	**RT**	**%E**	**RT**	**%E**
Cognates, initial	815.35 (22.30)	16.18 (1.85)	791.25 (24.59)	16.29 (2.60)	803.3 (23.40)	16.23 (2.24)
Cognates, second	901.02 (28.38)	30.45 (2.26)	888.08 (24.43)	31.25 (2.52)	894.55 (26.32)	30.85 (2.38)
Cognates, middle	888.12 (26.97)	25.40 (2.43)	864.59 (21.95)	24.17 (2.53)	876.35 (24.50)	24.78 (2.46)
Cognates, penultimate	935.81 (30.39)	46.75 (3.21)	877.98 (23.71)	26.90 (2.06)	906.89 (27.49)	36.82 (3.14)
Cognates, last	903.62 (25.34)	24.79 (2.71)	868.90 (25.82)	26.81 (2.8)	886.26 (25.57)	25.80 (2.74)
Non-cognates	742.69 (19.56)	8.09 (1.12)	720.18 (18.38)	6.69 (1.15)	731.43 (18.94)	7.39 (1.13)

*Standard errors are presented in parentheses*.

Data were analyzed using ANOVAs. Alpha was set to 0.05 for all analyses, and multiple comparisons were Bonferroni corrected. As in Experiment 1, two analyses were conducted: The first one examined the cognate status effect (i.e., if there were differences between cognates and non-cognates). The second one was restricted to cognate words and examined the critical question at stake (i.e., if cognate words with an internal deviant letter are worse recognized than cognates with the external or middle deviant letter, especially the first letter, revealing an inverted W-shaped function typically observed in the monolingual domain).

#### Cognate Status

In the first analysis, target words were analyzed using a cognate status (cognate or non-cognate) × language cue (Catalan or Spanish) design. In the analysis by participants, both factors were treated as a within-group factor, whereas in the analysis by items, language cue was treated as a within-group factor and cognate status was included as a between-group factor. ANOVA showed a main effect of cognate status: participants took longer time and committed more errors in discriminating cognates (mean RTs = 868 ms, mean %*E* = 26.90%) than non-cognates (mean RTs = 731 ms, mean %*E* = 7.39%), *F*_1(1, 36)_ = 144.12, MSE = 690,675, *p* < 0.001, *η*_*p*_^2^ = 0.80, *F*_2(1, 478)_ = 269.64, MSE = 4,701,425, *p* < 0.001, *η*_*p*_^2^ = 0.36, and *F*_1(1, 36)_ = 369.93, MSE = 14,079, *p* < 0.001, *η*_*p*_^2^ = 0.91, *F*_2(1, 478)_ = 310.14, MSE = 92,550, *p* < 0.001, *η*_*p*_^2^ = 0.40, for RT and errors, respectively. This result was expected as cognate words share the form, and hence discriminating them is more difficult than discriminating non-cognate words. In addition, a main effect of language cue was found, as Spanish cues (mean RTs = 787 ms, mean %*E* = 15.88%) were identified fastly and with less errors than Catalan cues (mean RTs = 813 ms, mean %*E* = 18.40%), *F*_1(1, 36)_ = 20.57, MSE = 25,769, *p* < 0.001, *η*_*p*_^2^ = 0.36, *F*_2(1, 478)_ = 10.60, MSE = 131,282, *p* = 0.001, *η*_*p*_^2^ = 0.02, and *F*_1(1, 36)_ = 5.56, MSE = 235.02, *p* = 0.024, *η*_*p*_^2^ = 0.13, *F*_2(1, 478)_ = 6.28, MSE = 1,467, *p* = 0.013, *η*_*p*_^2^ = 0.01, for RT and errors, respectively. No interaction was observed between cognate status and language (all *p*s > 0.05).

Although the faster and more precise responses to Spanish cues compared to Catalan cues led to an unexpected result because Spanish was the language labeled as the less preferred one, one plausible explanation comes up. Because Catalan was indeed a preferred language by the participants, Catalan cues may have provided more activation to their Spanish translations than the other way around. Therefore, Spanish words behaved as better competitors hampering the posterior identification of Catalan words.

#### Deviant Letter Position

**Figure 5:**
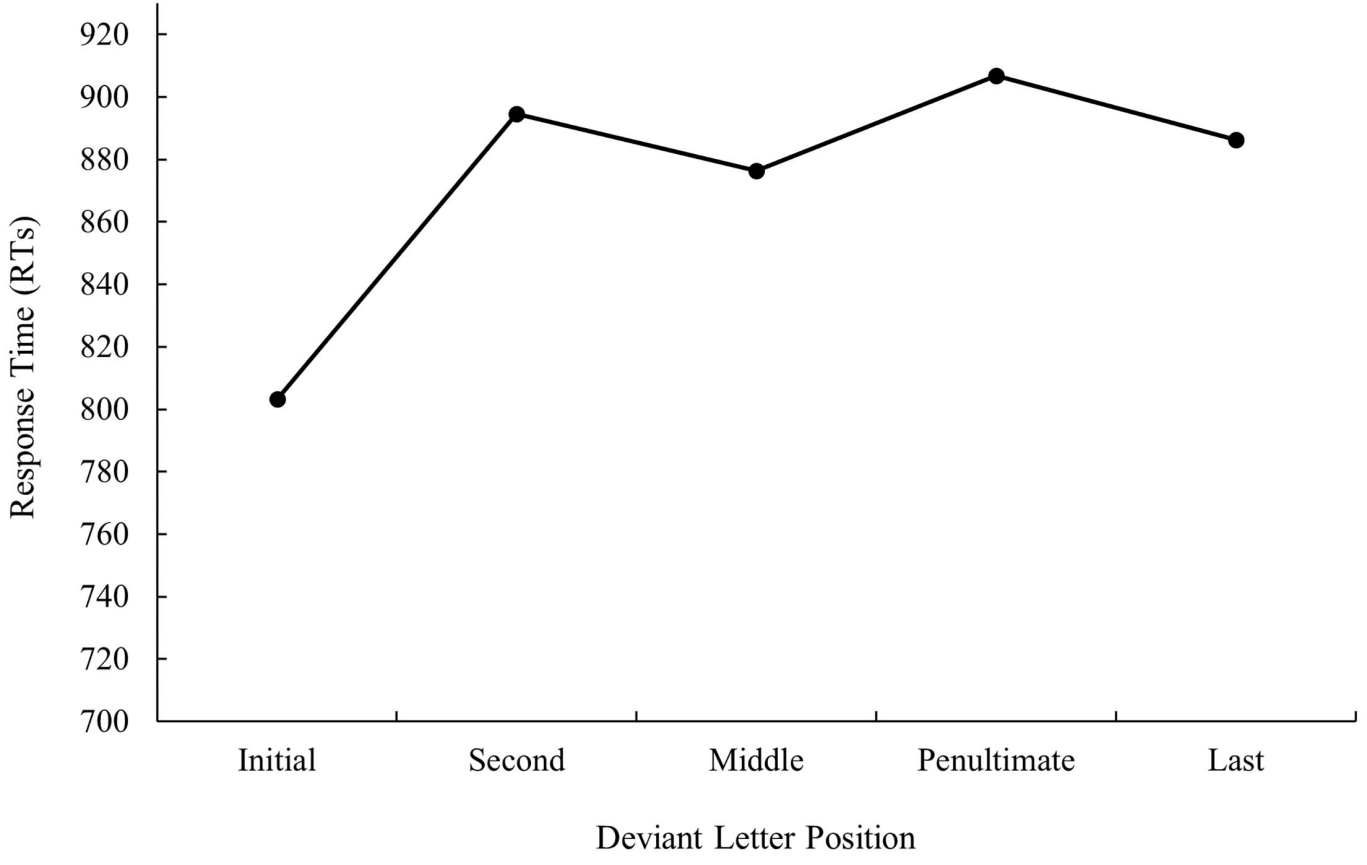
Mean response times (RTs; in ms) for cognate words according to its deviant letter position in Experiment 2a.

In the second analysis, we compared cognate words, which differ in the position of their deviant letter, with a deviant letter position (first, second, middle, penultimate, or last) × language cue (Catalan or Spanish) design. In the analysis by participants, both factors were within-group, whereas in the analysis by items, language cue was treated as a within-group factor and deviant letter position was included as a between-group factor. ANOVA on RTs yielded a main effect of deviant letter position, *F*_1(4, 144)_ = 11.02, MSE = 123,154, *p* < 0.001, *η*_*p*_^2^ = 0.23, *F*_2(4, 235)_ = 9.96, MSE = 158,700, *p* < 0.001, *η*_*p*_^2^ = 0.1 as cognate words with the deviant letter in the first position were identified faster than the rest of cognate words (all *p*s < 0.005) (see Figure 5).

**Figure 6:**
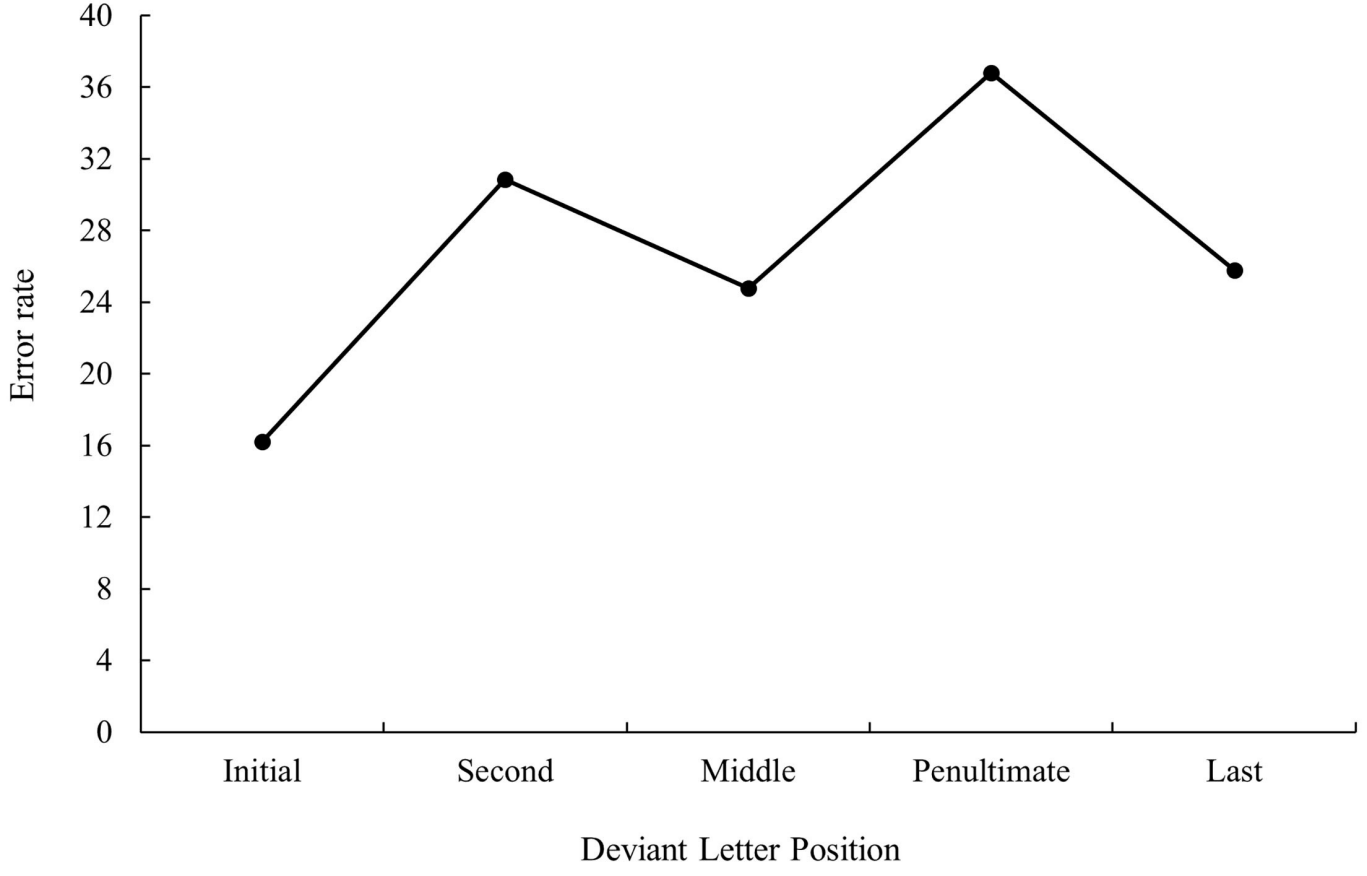
Mean error rate (in percentage) for cognate words according to its deviant letter position in Experiment 2a.

Similarly, the effect of deviant letter position was also significant in the analysis of error data, *F*_1(4, 144)_ = 22.02, MSE = 4,321, *p* < 0.001, *η*_*p*_^2^ = 0.38, *F*_2(4, 235)_ = 13.22, MSE = 5,458, *p* < 0.001, *η*_*p*_^2^ = 0.78. Pairwise comparisons showed that cognate words with the deviant letter in the first position were identified more precisely than cognate words in the other four conditions (all *p*s < 0.05). In addition, the identification of cognate words with the deviant letter in the penultimate position had more errors than cognate words with the deviant letter in any other position (all *p*s < 0.05; although the comparison between the penultimate and second position was not significant in the analysis by items). As it can be seen from Figures 5, 6, these results showed an expected inverted W-shaped function both in the latency and error data and, therefore, replicate the findings observed in the monolingual domain (see Gómez et al., 2008).

In addition, the results showed a main effect of language cue, both in the latency, *F*_1(1, 36)_ = 10.67, MSE = 86,742, *p* = 0.002, *η*_*p*_^2^ = 0.23, *F*_2(1, 235)_ = 4.75, MSE = 76,343, *p* = 0.03, *η*_*p*_^2^ = 0.02, and error data, *F*_1(1, 36)_ = 3.91, MSE = 1,219, *p* = 0.056, *η*_*p*_^2^ = 0.10, *F*_2(1, 235)_ = 4.26, MSE = 1,477, *p* = 0.04, *η*_*p*_^2^ = 0.02. Spanish cues (mean RTs = 858 ms, mean %*E* = 25.08%) were identified faster and with less errors than Catalan cues (mean RTs = 889 ms, mean %*E* = 28.71%). Furthermore, the interaction between deviant letter position and language cue reached significance in the analysis of error data, *F*_1(4, 144)_ = 10.73, MSE = 1,547, *p* < 0.001, *η*_*p*_^2^ = 0.23, *F*_2(4, 235)_ = 5.70, MSE = 1,979, *p* < 0.001, *η*_*p*_^2^ = 0.09. Pairwise comparisons revealed that cognate words with the deviant letter in the penultimate position showed a different pattern of error rates between languages. Specifically, Catalan cues with the deviant letter in the penultimate position were identified with more errors than cognate words in the other four conditions, whereas Spanish cues with the deviant letter in the penultimate position did not differ in the rest of conditions. Although this result is interesting and potentially reveal a differential processing in the way of coding letter position during the recognition of Catalan and Spanish words by bilinguals, the truth is that the pattern of results was pretty similar in both languages (i.e., an inverted W-shaped function; see Table 7). The fact that participants had been faster and more precise when the cue was presented in Spanish, possibly attenuating the differences across conditions. This would indicate that Spanish words worked as better discriminating cues than Catalan words probably because they provide less activation to their Catalan translations as we mentioned before. If this is the case, then a control group of native speakers of Spanish with no knowledge of Catalan should show a more robust effect of deviant letter position when the cues are presented in Catalan. Note that for these participants, the Catalan cues would be non-words, and hence no sign of lexical interference from these cues to Spanish words would be expected. In other words, an activation from non-word cues to words would be higher than the other way around as words never activate non-words. To examine this issue, Experiment 2b was conducted with a group of Spanish monolinguals.

**Table 7:** Mean response times (RTs; in milliseconds) and percentage of errors (% E) in the different experimental conditions of Experiment 2b.

	**Catalan cue**		**Spanish cue**		**Total**	
	**RT**	**%E**	**RT**	**%E**	**RT**	**%E**
Cognates, initial	841.59 (22.01)	23.66 (2.79)	794.1 (22.26)	16.55 (2.68)	817.84 (22.03)	20.11 (2.75)
Cognates, second	912.89 (29.22)	43.95 (3.44)	881.63 (21.32)	22.99 (2.73)	897.26 (25.08)	33.47 (3.64)
Cognates, middle	892.73 (26.28)	33.44 (4.62)	865.33 (29.50)	19.47 (2.43)	879.03 (27.34)	26.45 (3.84)
Cognates, penultimate	954.02 (41.40)	54.25 (3.85)	862.41 (32.08)	18.40 (2.37)	908.22 (37.13)	36.32 (4.67)
Cognates, last	873.35 (34.04)	30.00 (4.15)	856.92 (28.38)	17.96 (2.74)	865.13 (30.54)	23.98 (3.62)
Non-cognates	786.88 (23.26)	9.94 (1.36)	716.94 (20.03)	5.19 (0.87)	751.91 (22.19)	7.56 (1.20)

*Standard errors are presented in parentheses*.

## Experiment 2B

### Method

#### Participants

About 32 native speakers of Spanish (28 women and 4 men, mean age 21.47 years, SD = 2.02) participated in the experiment in exchange for academic credits. None of the participants took part in any of the previous experiments. They were undergraduate students from the University of Granada (Granada, Spain). Participants were asked to fill in a questionnaire similar to that of Experiment 1, in which they had to rate their ability in several languages (i.e., Spanish, English, French, and Catalan) in listening, speaking, reading, and writing by using a seven-point Likert scale (1 = “very poor” in the assessed skill, 7 = “native”). According to the ratings of the questionnaire, none of the participants had knowledge of Catalan. The fluency in Spanish of participants is reported in Table 5.

#### Materials and Procedure

The materials and procedure are the same as those used in Experiment 2a.

### Results

The data from the four participants with more than 30% of the errors (each from a different experimental version of the experiment) were discarded from analyses. In addition, RTs faster than 250 ms or slower than 1,600 ms were removed (<5% of the whole). Then, we calculated the mean RTs for the correct responses and the mean %*E* across experimental conditions (see Table 7). We conducted the analyses similar to those in Experiment 2a.

#### Cognate Status

The results showed a main effect of cognate status, indicating that participants took longer and did more errors in identifying cognates (mean RTs = 875 ms; mean %*E* = 28.07%) than non-cognates due to their form overlap (mean RTs = 752 ms; mean %*E* = 7.56%), *F*_1(1, 27)_ = 278.11, MSE = 421,479, *p* < 0.001, *η*_*p*_^2^ = 0.91, *F*_2(1, 476)_ = 46.15, MSE = 69,080, *p* < 0.001, *η*_*p*_^2^ = 0.09, and *F*_1_(1, 27) = 226.28, MSE = 11,771, *p* < 0.001, *η*_*p*_^2^ = 0.89, *F*_2(1, 478)_ = 347.38, MSE = 99,750, *p* < 0.001, *η*_*p*_^2^ = 0.42, for RT and errors, respectively. A main effect of language cue was also observed: Spanish cues (mean RTs = 785 ms, mean %*E* = 12.13%) were identified faster and with less errors than Catalan (and thus non-word) cues (mean RTs = 842 ms, mean %*E* = 23.5%), *F*_1(1, 27)_ = 44.27, MSE = 92,531, *p* < 0.001, *η*_*p*_^2^ = 0.62, *F*_2(1, 476)_ = 46.15, MSE = 690,793, *p* < 0.001, *η*_*p*_^2^ = 0.09, and *F*_1(1, 27)_ = 41.11, MSE = 3,618, *p* < 0.001, *η*_*p*_^2^ = 0.6, *F*_2(1, 478)_ = 106.85, MSE = 29,492, *p* < 0.001, *η*_*p*_^2^ = 0.18, for RT and errors, respectively, probably because of the effect of lexicality as only Spanish cues were words for these participants. In addition, an interaction between cognate status and language was found in error data, *F*_1__(1, 27)_ = 35.99, MSE = 1,226, *p* < 0.001, *η*_*p*_^2^ = 0.57, *F*_2(1, 478)_ = 32.19, MSE = 8,885, *p* < 0.001, *η*_*p*_^2^ = 0.06. This interaction showed that, although the effect of language appeared for cognate and non-cognates, the effect was larger for the former (mean %*E* = 17.98 and 4.75%, respectively, all *p*s < 0.05).

#### Deviant Letter Position

**Figure 7:**
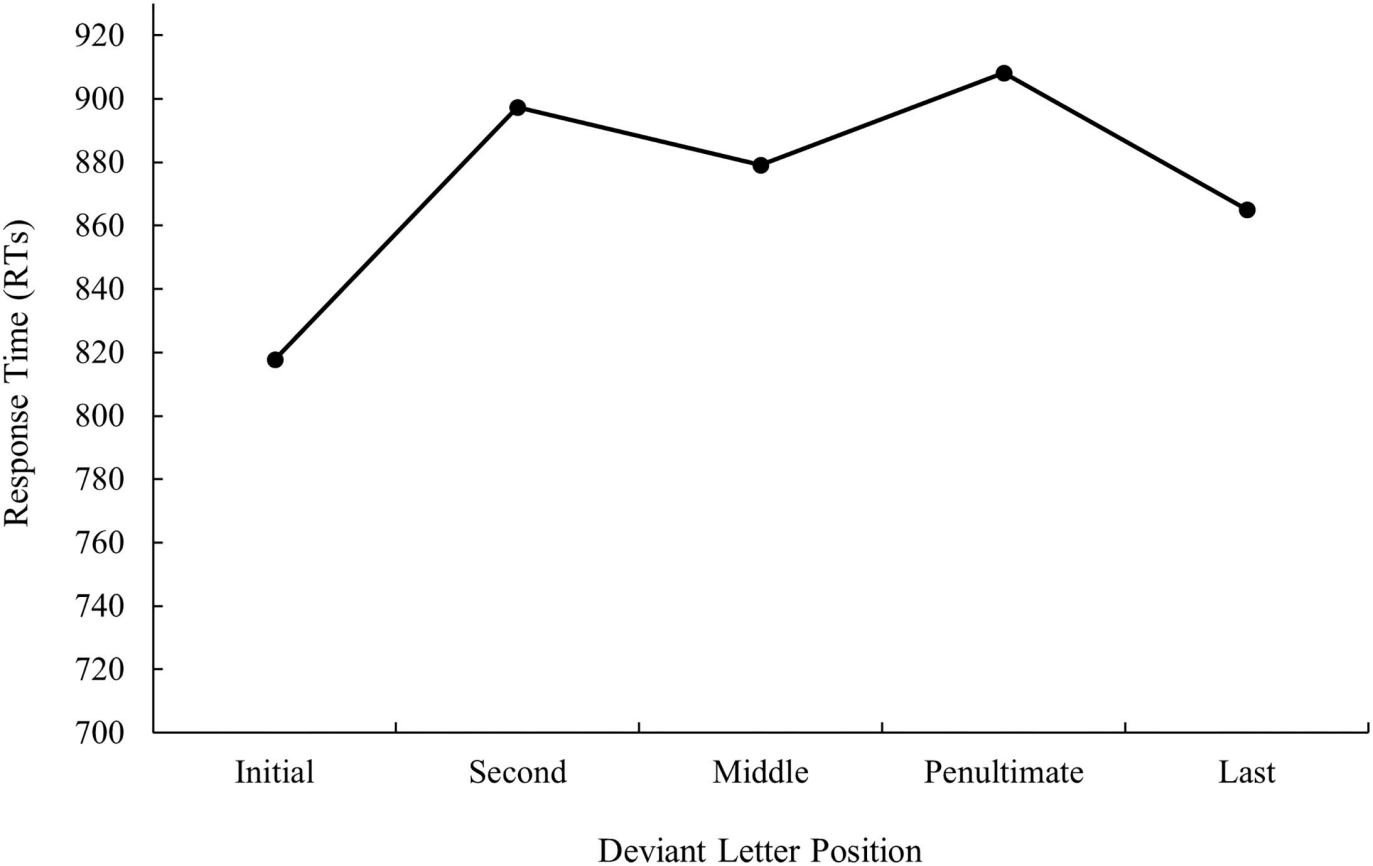
Mean response times (RTs; in ms) for cognate words according to its deviant letter position in Experiment 2b.

ANOVA on latency data showed a main effect of deviant letter position, *F*_1(4, 104)_ = 9.11, MSE = 66,837, *p* < 0.001, *η*_*p*_^2^ = 0.26, *F*_2(4, 233)_ = 5.31, MSE = 111,897, *p* < 0.001, *η*_*p*_^2^ = 0.08. Translation pairs with the deviant letter in the first position were identified faster than those with the deviant letter in the other positions (all *p*s < 0.05) (see Figure 7).

**Figure 8:**
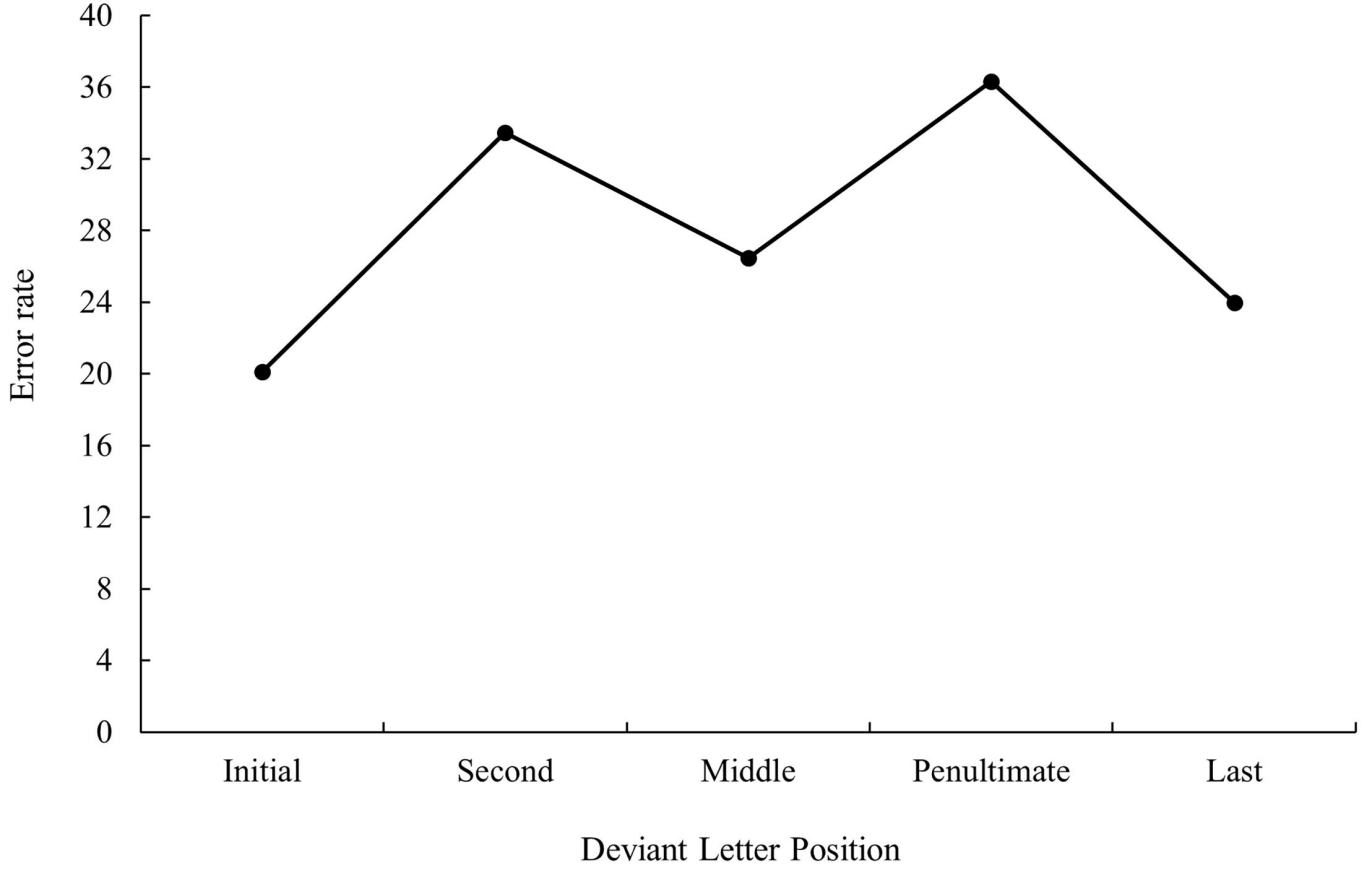
Mean error rate (in percentage) for cognate words according to its deviant letter position in Experiment 2b.

The effect of deviant letter position was also significant in the analysis of error data, *F*_1(4, 108)_ = 14.13, MSE = 2,520, *p* < 0.001, *η*_*p*_^2^ = 0.34, *F*_2(4, 235)_ = 10.19, MSE = 4,081, *p* < 0.001, *η*_*p*_^2^ = 0.15. Translation pairs with the deviant letter in the penultimate position were identified with more errors than those with the deviant letter in the first, middle, and last position (all *p*s < 0.05). In addition, translation pairs with the deviant letter in the second position were identified with more errors than those with the deviant letter in the first and last position (all *p*s < 0.05) (see Figure 8).

Finally, a main effect of language cue was found, both in the latency, *F*_1(1, 26)_ = 13.56, MSE = 106,672, *p* = 0.001, *η*_*p*_^2^ = 0.34, *F*_2(1, 233)_ = 9.91, MSE = 199,288, *p* = 0.002, *η*_*p*_^2^ = 0.04, and error data, *F*_1(1, 27)_ = 43.1, MSE = 22,640, *p* < 0.001, *η*_*p*_^2^ = 0.61, *F*_2(1, 235)_ = 93.85, MSE = 35,375, *p* < 0.001, *η*_*p*_^2^ = 0.29. Spanish cues (mean RTs = 852 ms, mean %*E* = 19.08%) were identified faster and with less errors than Catalan (non-word) cues (mean RTs = 897 ms, mean %*E* = 37.06%). In addition, and as expected, a significant interaction between deviant letter position and language cue was observed in the analysis of error data, *F*_1(4, 108)_ = 9.86, MSE = 1,742, *p* < 0.001, *η*_*p*_^2^ = 0.27, *F*_2(4, 235)_ = 7.69, MSE = 2,900, *p* < 0.001, *η*_*p*_^2^ = 0.12. The effect of deviant letter position was only found with Catalan (and thus non-word) cues: Stimuli pairs with the deviant letter in the penultimate position were identified with more errors than those with the deviant letter in the first, middle, and last position (all *p*s < 0.05); in addition, stimuli pairs with the deviant letter in the second position were identified with more errors than those with the deviant letter in the first and last position (all *p*s < 0.05). It seems that the effect of deviant letter position is higher when there is less lexical competition. This finding is consistent with that observed by Lin and Lin (2016): the lesser the number of orthographic neighbors is the higher the transposition effect will be (see also the studies by Forster et al., 1987; Perea and Rosa, 2000; Kinoshita et al., 2009, in the monolingual domain).

## General Discussion

The present study was designed to test the input-coding scheme of the multilink model (Dijkstra et al., 2019) by manipulating the deviant letter position of Catalan–Spanish cognate words. The most commonly employed tasks in the study of letter position coding during word recognition were used: The masked priming lexical decision task (Experiments 1a and 1b), and the 2AFCT (Experiments 2a and 2b). For the sake of simplicity, the multilink assumes that the positions of the letters in a word are established very early in processing, and hence no letter position has a special role over the others. The findings of the experiments presented here with Catalan–Spanish bilinguals who refuse this tenet showing that letters occupying the first position are preferentially processed in comparison to letters in any other position (Experiments 1a and 2a) as it occurs with monolinguals during word recognition (Experiment 1b and 2b). Therefore, the mechanism used by bilinguals to code letter position information seems to be similar to that used by monolinguals, at least when the two languages are alphabetic and were acquired early in life (see Yang et al., 2021 for the differences across languages with a different script). The privileged role of the first letter over the others during word recognition is reflected in the most influential (and more flexible) input-coding schemes developed in the monolingual domain, such as the overlap model (Gómez et al., 2008), the SERIOL model (Whitney, 2001), or the SOLAR model (Davis, 1999). Thus, for instance, in the overlap model, the estimated similarity between Catalan–Spanish cognate words that vary in their first-letter positions is lower than the estimated similarity for cognate words whose deviant letter is in any other position (e.g., 1.14 for xifra-cifra [number], 1.57 for llebre-liebre [hare], 1.59 for ploma-pluma [feather], 1.69 for dansa-danza [dance], and 1.70 for rostre-rostro [face]). These values were calculated by considering the parameters reported in Gómez et al. (2008, Experiment 1). Similarly, in the SOLAR model, the estimated similarity between the cognate words that vary in their first-letter position is lower than for the other cognate conditions (e.g., 0.71 for xifra-cifra [number], 0.88 for llebre-liebre [hare], 0.86 for ploma-pluma [feather], 0.86 for dansa-danza [dance], and 0.75 for rostre-rostro [face]). These values were obtained from Colin Davis' Match Calculator application (available at http://www.pc.rhul.ac.uk/staff/c.davis/Utilities/MatchCalc/).

The first-letter advantage observed in bilinguals (especially in Experiment 2 with the 2AFCT) and monolinguals suggests that initial letter is the most informative one regarding word identity. Although *a priori* we could think that these findings reflect an early sequential, beginning-to-end, orthographic processing, a result appears to rule out this hypothesis: The inverted W-shaped function observed in the 2AFCT instead of a linear trend. Indeed, although the differences across cognate conditions have reached a significance on latency and error data only when considering the first-letter word, the absence of a last-letter advantage on latency may have been due to the cue duration used (50 ms). As pointed out by Whitney (2001), whereas studies showing a final-letter advantage involved presentation durations ≥75 ms, those in which a final-letter advantage did not occur involved presentation durations of 50 ms or less. In this field of study, Gómez et al. (2008) also failed to observe the final-letter advantage using a cue duration of 60 ms. The first-letter advantage is, therefore, more robust and can be, as per Tydgat and Grainger (2009), due to the changes in the size and shape of receptive fields (more elongated receptive fields to first-letter detectors). These changes would arise to optimize processing at the first-letter position, at least in Roman alphabetic languages (the languages in which the first-letter advantage was observed). This is because first letters provide more constraints on lexical identity and are also critical for translating an orthographic code into a phonological one (note that correct graphemes can be computed only with precise order information). In addition, Johnson et al. (2007) suggest that the identification of the initial letter but not the other letters of a word may be dependent on the absolute letter position. Whatever be the underlying mechanism responsible for such preferential processing, what is clear is that the input-coding scheme of the multilink model should accommodate these findings as well as other empirical evidence with bilinguals (Witzel et al., 2011; Lin and Lin, 2016; Yang et al., 2021), by assuming either a certain degree of perceptive noise when assigning letters to positions (similar to the overlap model developed in the monolingual domain (Gómez et al., 2008) or the activation of open bigrams (see, for instance, Grainger et al., 2006).

We recognize, however, that more research considering different languages as well as more bilingual populations (e.g., balanced, unbalanced, and speaking languages with more or less similar scripts) is needed before implementing a new input scheme in the multilink model as some modulations in letter position coding were found as a function of language (more or less preferred). Indeed, even when lexical word frequencies were matched across languages, the responses in the 2AFCT were faster and more precise with the less preferred language (Spanish) cue words. Besides and more important, the effect of deviant letter position seemed to be more robust when the cue led to a lesser degree of lexical competition (Spanish cues in Experiment 2a and Catalan cues in Experiment 2b). Because Catalan is a preferred language by the participants, Catalan words could have provided more activation to their Spanish translations. As a consequence, lexical competition between the two-word readings highly reflected a greater confusability in word identification. As stated by the multilink model, this is consistent with the idea of an asymmetrical cross-linguistic cognate activation in sequential bilinguals, which can be extended to early bilinguals whenever they make a differential usage of the spoken languages. In other words, compared to their Catalan counterparts, Spanish words may work as better cues for word identification. This seems to be the case because cues are acting as non-words (the Catalan words for the Spanish monolinguals from Experiment 2b), the effect of deviant letter position is robust. Similarly, if we had used higher frequency values of cue Spanish words with bilinguals, it would be expected a greater competition in a similar way as happening in the monolingual domain with neighbor words (see Perea, 1998).

Another important issue for future research is to examine in further detail to what extent the flexibility of the orthographic coding scheme depends on the different languages known by a bilingual person as well as on the degree of L2 proficiency. This is because, whereas the first-letter advantage appeared in Roman alphabetic languages, it does not appear in other alphabetic languages like Thai (see Perea et al., 2011; Winskel et al., 2012; Yang et al., 2021). Thus, for instance, Perea et al. (2011) stated the characteristics of Thai leading to the actual identity of the letter being more critical than letter position. Indeed, the authors observed that letter position encoding in this language is relatively flexible due to the existence of certain flexibility in the ordering of the letters (it does not necessarily correspond to the ordering of phonemes of a word) and the lack of inter-word spaces. These language features create a certain level of ambiguity in relation to the demarcation of word boundaries (see Perea et al., 2011; Winskel et al., 2012), which would explain the absence of first-letter advantage. Thus, it would be interesting to examine letter position coding in learning to read an L2, which has different characteristics from L1. For instance, the study of letter position coding during L2 word recognition with the bilinguals of Thai and English with different degrees of L2 proficiency would enable researchers to examine to what extent the characteristics of different languages as well as the degree of L2 proficiency shape the way of coding letter position. As a consequence, the properties of the visual word recognition system that are specific to a given script would be disentangled.

To summarize, the present research strongly suggests that the mechanisms underlying letter position coding are similar in case of bilinguals and monolinguals, at least when bilinguals speak alphabetic languages in which a privileged role of the first letters over the others is observed. Some modulations were, however, observed as a function of language cue in the 2AFCT as Spanish cue words led to faster and more precise responses than their Catalan counterparts, probably due to a different degree of lexical competition provided by the cues. Overall, the findings suggest that the input-coding scheme of the multilink model should be amended.

## Data Availability Statement

The datasets presented in this study can be found in online repositories. The names of the repository/repositories and accession number(s) can be found at: https://osf.io/mqhu5/?view_only=94cdf0d86e0b4e7e8b2757e33f6a78ce.

## Ethics Statement

The studies involving human participants were reviewed and approved by The Ethics Council of the University of Minho (CEICSH 023/2014). The patients/participants provided their written informed consent to participate in this study.

## Author Contributions

MC conceived the idea and experimental design of the study and was responsible for writing and editing the manuscript overall. PF, JH, and PM contributed to the main theoretical hypotheses and interpretations. JH and PM managed the data collection. JH analyzed the data and wrote the results sections of the paper. All authors reviewed the different drafts of the manuscript and made theoretical contributions for the general discussion.

## Funding

This study was conducted at the Psychology Research Center (CIPsi/UM) School of Psychology, University of Minho, supported by the Foundation for Science and Technology (FCT) through the Portuguese State Budget (UIDP/01662/2020). This was also funded by the Spanish Ministry of Economy and Competitiveness (PCIN-2015-165-C02-02 and MINECO/FEDER), by the Spanish Ministry of Science, Innovation and Universities (RED2018-102615-T), and by the Research Promotion Program of the Universitat Rovira I Virgili (2018PFR-URV-B2-32).

## Conflict of Interest

The authors declare that the research was conducted in the absence of any commercial or financial relationships that could be construed as a potential conflict of interest.

## Publisher's Note

All claims expressed in this article are solely those of the authors and do not necessarily represent those of their affiliated organizations, or those of the publisher, the editors and the reviewers. Any product that may be evaluated in this article, or claim that may be made by its manufacturer, is not guaranteed or endorsed by the publisher.

## Footnotes

^1^It should nevertheless be noted that whereas the first-position advantage for letter strings is a robust phenomenon, the middle-position and final-position advantage has not always been observed and seems to be modulated by task requirements (see Tydgat and Grainger, 2009, for more details).

^2^In addition to ANOVAs, we also analyzed the data of the four experiments by means of linear mixed-effects models. The results followed the same pattern like ANOVAs. The data and scripts used for the linear mixed-effect analyses are available in the following link: https://osf.io/mqhu5/?view_only=94cdf0d86e0b4e7e8b2757e33f6a78ce.

^3^We initially analyzed the data for monolinguals and bilinguals within the same model. The results barely differ from the separate analysis. However, there are some subtle differences between the pattern of results from the two groups, which complicate the presentation, reading, and interpretation of the results when the data from both groups are presented in the same model. This is mainly due to the presence of second- and third -order interactions, which can be somewhat difficult to interpret. Therefore, for the sake of clarity, we have decided to present the results individually for each language group.
