# Corrigendum: Disentangling the role of deviant letter position on cognate word processing

**DOI:** 10.3389/fpsyg.2022.1091796

**Published:** 2022-11-28

**Authors:** Montserrat Comesaña, Juan Haro, Pedro Macizo, Pilar Ferré

**Affiliations:** ^1^Research Unit in Human Cognition, CIPsi, School of Psychology, University of Minho, Braga, Portugal; ^2^Center for Cognitive Science (C3), Nebrija University, Madrid, Spain; ^3^Department of Psychology and CRAMC, Universitat Rovira I Virgili, Tarragona, Spain; ^4^Department of Experimental Psychology and Mind, Brain and Behavior Research Center (CIMCYC), University of Granada, Granada, Spain

**Keywords:** letter-position encoding, cognate recognition, multilink, masked priming lexical decision task, 2-alternative forced-choice task

In the published article, there was an error in the wording of the **Funding** statement. The Funding statement previously stated: “This study was conducted at the Psychology Research Center (CIPsi/UM), School of Psychology, University of Minho, supported by the Foundation for Science and Technology (FCT) and FEDER through COMPETE2020 under the PT2020 Partnership Agreement POCI-01-0145-FEDER-007653. This was been also funded by the Spanish Ministry of Economy and Competitiveness (PCIN-2015-165-C02-02 and MINECO/FEDER), by the Spanish Ministry of Science, Innovation and Universities (RED2018-102615-T), and by the Research Promotion Program of the Universitat Rovira i Virgili (2018PFR-URV-B2-32).”

The corrected **Funding** statement appears below:

This study was conducted at the Psychology Research Center (CIPsi/UM) School of Psychology, University of Minho, supported by the Foundation for Science and Technology (FCT) through the Portuguese State Budget (UIDP/01662/2020). This was also funded by the Spanish Ministry of Economy and Competitiveness (PCIN-2015-165-C02-02 and MINECO/FEDER), by the Spanish Ministry of Science, Innovation and Universities (RED2018-102615-T), and by the Research Promotion Program of the Universitat Rovira I Virgili (2018PFR-URV-B2-32).

The authors apologize for this error and state that this does not change the scientific conclusions of the article in any way. The original article has been updated.

## Publisher's note

All claims expressed in this article are solely those of the authors and do not necessarily represent those of their affiliated organizations, or those of the publisher, the editors and the reviewers. Any product that may be evaluated in this article, or claim that may be made by its manufacturer, is not guaranteed or endorsed by the publisher.

