# Supplementary material for: Disentangling the Role of Deviant Letter Position on Cognate Word Processing
Source: Front Psychol. 2021 Sep 24;12:731312. doi: 10.3389/fpsyg.2021.731312 (PMC8497793; doi:10.3389/fpsyg.2021.731312)
Supplement: Supplementary file 1 [file Data_Sheet_1.pdf]

Appendix. Spanish and Catalan translations (and their English translations) used in the Experiments 1 and 2. CG and NCG stand for cognate and non-cognate words respectively. Cognate words are organized per experimental condition (i.e., as a function of the location of their deviant letter: initial, second, middle, penultimate and last).

Condition	Target - Spanish	Target - Catalan	English Translation	Catalan Control	English Translation
CG_initial	Vasca	basca	*Basque*	ungla	*nail*
CG_initial	Otorgar	atorgar	*to grant*	engolir	*to swallow*
CG_initial	Hilar	filar	*to spin*	bufar	*to blow*
CG_initial	Cifra	xifra	*number*	lliçó	*lesson*
CG_initial	Cuota	quota	*share*	allau	*avalanche*
CG_initial	Helada	gelada	*frost*	brusca	*abrupt*
CG_initial	Cuarta	quarta	*quarter*	futura	*future*
CG_initial	Celosa	gelosa	*jealous*	regada	*watered*
CG_initial	Cuadra	quadra	*block*	estufa	*stove*
CG_initial	Higuera	figuera	*fig tree*	adopció	*adoption*
CG_initial	Inglesa	anglesa	*english*	curiosa	*curious*
CG_initial	Herir	ferir	*to hurt*	picar	*itch*
CG_initial	Harina	farina	*flour*	camisa	*shirt*
CG_initial	Hermana	germana	*sister*	lectura	*reading*
CG_initial	Hoguera	foguera	*campfire*	mainada	*children*
CG_initial	Evaluar	avaluar	*to evaluate*	crèixer	*grow up*
CG_initial	Hormiga	formiga	*ant*	barbeta	*chin*
CG_initial	Enfermera	infermera	*nurse*	perifèria	*periphery*
CG_initial	Herradura	ferradura	*horseshoe*	carabassa	*pumpkin*
CG_initial	Llena	plena	*full*	dreta	*right*
CG_initial	llorar	plorar	*to cry*	néixer	*to be born*
CG_initial	madrina	padrina	*godmother*	estrofa	*stanza*
CG_initial	llanura	planura	*plain*	caverna	*cave*

CG_initial	llamarada	flamarada	*flare*	opulència	*opulence*
CG_second	tumbar	tombar	*lay down*	ploure	*to rain*
CG_second	tumba	tomba	*tomb*	drama	*drama*
CG_second	rumor	remor	*rumor*	ràdio	*radio*
CG_second	pelota	pilota	*ball*	estada	*stay*
CG_second	surgir	sorgir	*to arise*	elevar	*lift up*
CG_second	pulsera	polsera	*bracelet*	carreta	*cart*
CG_second	soportar	suportar	*to support*	preveure	*foresee*
CG_second	suspirar	sospirar	*to sigh*	concebir	*to conceive*
CG_second	inmensa	immensa	*immense*	perduda	*lost*
CG_second	ejecutar	executar	*to run*	reclamar	*to claim*
CG_second	explanada	esplanada	*esplanade*	tovallola	*towel*
CG_second	inmediata	immediata	*immediate*	conscient	*conscious*
CG_second	ayuda	ajuda	*help*	calma	*calm*
CG_second	blusa	brusa	*blouse*	farsa	*farce*
CG_second	cubrir	cobrir	*to cover*	pregar	*pray*
CG_second	cumplir	complir	*to achieve*	dirigir	*direct*
CG_second	inmortal	immortal	*immortal*	ignorant	*ignorant*
CG_second	inmutable	immutable	*immutable*	preferent	*preferable*
CG_second	montada	muntada	*assembled*	dictada	*dictated*
CG_second	montura	muntura	*mount*	cotorra	*parrot*
CG_second	isla	illa	*island*	rosa	*pink*
CG_second	nadar	nedar	*to swim*	calar	*shut up*
CG_second	montar	muntar	*to mount*	trucar	*to call*
CG_second	liebre	llebre	*hare*	crosta	*crust*
CG_middle	rezar	resar	*pray*	criar	*to breed*
CG_middle	valiosa	valuosa	*valuable*	errònia	*wrong*
CG_middle	pluma	ploma	*feather*	canal	*channel*
CG_middle	prensa	premsa	*press*	pèrdua	*loss*

CG_middle	vengar	venjar	*to avenge*	copiar	*to copy*
CG_middle	tarjeta	targeta	*card*	bastida	*scaffolding*
CG_middle	haber	haver	*to have*	donar	*to give*
CG_middle	bolsa	bossa	*bag*	faixa	*corset*
CG_middle	cabida	cabuda	*room*	barata	*cheap*
CG_middle	bailar	ballar	*to dance*	pintar	*to paint*
CG_middle	calzada	calçada	*road*	asperesa	*roughness*
CG_middle	forzada	forçada	*forced*	cantada	*sung*
CG_middle	forzosa	forçosa	*mandatory*	alterada	*altered*
CG_middle	enfrentar	enfrontar	*to deal*	encomanar	*to entrust*
CG_middle	cazar	caçar	*to hunt*	ofegar	*to drown*
CG_middle	bazar	basar	*bazaar*	botxí	*a little bit of*
CG_middle	cobre	coure	*copper*	museu	*museum*
CG_middle	copla	cobla	*folk song*	funda	*cover*
CG_middle	cisne	cigne	*swan*	àlbum	*album*
CG_middle	capilla	capella	*chapel*	reacció	*reaction*
CG_middle	escupir	escopir	*to spit*	diferir	*to differ*
CG_middle	descubrir	descobrir	*to discover*	convertir	*to transform*
CG_middle	cartulina	cartolina	*cardboard*	ballarina	*dancer*
CG_middle	mayor	major	*larger*	ordre	*order*
CG_penultimate	renta	renda	*to rent*	cinta	*tape*
CG_penultimate	plaza	plaça	*square*	túnica	*robe*
CG_penultimate	venta	venda	*sale*	marca	*mark*
CG_penultimate	pureza	puresa	*purity*	enveja	*envy*
CG_penultimate	retener	retenir	*to hold back*	adreçar	*to contact*
CG_penultimate	danza	dansa	*dance*	broma	*joke*
CG_penultimate	avanzar	avançar	*to progress*	reposar	*to rest*
CG_penultimate	atrever	atrevir	*dare*	invocar	*to invoke*
CG_penultimate	destreza	destresa	*skill*	deshonra	*dishonor*

CG_penultimate	compleja	complexa	*complex*	imperial	*imperial*
CG_penultimate	contener	contenir	*to contain*	retornar	*to return*
CG_penultimate	calva	calba	*bald spot*	àcida	*acid*
CG_penultimate	coser	cosir	*to sew*	segar	*harvest*
CG_penultimate	atlas	atles	*atlas*	amoni	*ammonium*
CG_penultimate	aclarar	aclarir	*clear out*	iniciar	*to start*
CG_penultimate	ceguera	ceguesa	*blindness*	solista	*soloist*
CG_penultimate	certeza	certesa	*certainty*	higiene	*hygiene*
CG_penultimate	cerveza	cervesa	*beer*	tristor	*sadness*
CG_penultimate	agudeza	agudesa	*acuity*	cascada	*waterfall*
CG_penultimate	conceder	concedir	*to grant*	allargar	*lengthen*
CG_penultimate	descender	descendir	*to descend*	delimitar	*to delimit*
CG_penultimate	joya	joia	*jewel*	raça	*race*
CG_penultimate	once	onze	*eleven*	pati	*courtyard*
CG_penultimate	mentira	mentida	*lie*	empenta	*push*
CG_last	rastro	rastre	*trail*	marbre	*marble*
CG_last	rostro	rostre	*face*	missió	*mission*
CG_last	pacto	pacte	*pact*	rigor	*rigour*
CG_last	rapto	rapte	*rapture*	clixé	*cliché*
CG_last	templo	temple	*temple*	senyal	*signal*
CG_last	perdiz	perdiu	*partridge*	anella	*ring*
CG_last	turismo	turisme	*tourism*	regidor	*councilor*
CG_last	astro	astre	*star*	pilot	*pilot*
CG_last	centro	centre	*center*	procés	*process*
CG_last	flan	flam	*flan*	ànec	*duck*
CG_last	correo	correu	*mail*	paquet	*package*
CG_last	ateneo	ateneu	*athenaeum*	jovent	*youth*
CG_last	diablo	diable	*devil*	retrat	*portrait*
CG_last	defecto	defecte	*defect*	torrent	*torrent*

CG_last	directo	directe	*direct*	descans	*rest*
CG_last	benigno	benigne	*benign*	ignorat	*ignored*
CG_last	feminismo	feminisme	*feminism*	venciment	*maturity*
CG_last	modo	mode	*mode*	disc	*disc*
CG_last	lucro	lucre	*profit*	dòlar	*dollar*
CG_last	matriz	matriu	*matrix*	fletxa	*arrow*
CG_last	majestad	majestat	*majesty*	salvador	*rescuer*
CG_last	abad	abat	*abbot*	codi	*code*
CG_last	abismo	abisme	*abyss*	traçat	*layout*
CG_last	optimismo	optimisme	*optimism*	dormitori	*bedroom*
NCG	yate	iot	*yacht*	mag	*mag*
NCG	vello	borrissol	*hair*	guanyador	*winner*
NCG	tomar	prendre	*to drink*	esperar	*to wait*
NCG	plazo	termini	*term*	vehicle	*vehicle*
NCG	tirar	llençar	*to throw*	incloure	*to include*
NCG	quitar	treure	*to remove*	quedar	*meet up*
NCG	pereza	mandra	*sloth*	jugada	*play*
NCG	rotura	trencament	*break*	termòmetre	*thermometer*
NCG	aceite	oli	*oil*	joc	*game*
NCG	vaciar	buidar	*to empty*	regnar	*to reign*
NCG	recreo	esbarjo	*escape*	festeig	*engagement*
NCG	volver	tornar	*to return*	pensar	*to think*
NCG	rendija	escletxa	*slit*	marquesa	*marquesa*
NCG	abanico	ventall	*fan*	clavell	*carnation*
NCG	tropiezo	ensopegada	*tumble*	despectiva	*contemptuous*
NCG	relámpago	llampec	*flash of lightning*	sinònim	*synonymous*
NCG	trasfondo	rerefons	*background*	abductor	*abductor*
NCG	tragar	empassar	*to swallow*	arriscar	*to risk*
NCG	red	xarxa	*net*	vinya	*vineyard*

NCG	queso	formatge	*cheese*	julivert	*parsley*
NCG	tonto	ximple	*fool*	genial	*great*
NCG	rubio	ros	*blond*	all	*garlic*
NCG	trigo	blat	*wheat*	text	*text*
NCG	pedir	demanar	*to ask*	obtenir	*get*
NCG	toldo	tendal	*awning*	pallol	*pallol*
NCG	parche	pegat	*patch*	guiat	*guided*
NCG	rebaño	ramat	*flock*	estel	*star*
NCG	pájaro	ocell	*bird*	carro	*trolley*
NCG	pierna	cama	*leg*	àvia	*grandmother*
NCG	relleno	farcit	*filling*	afinat	*refined*
NCG	sobrina	neboda	*niece*	esmena	*amendment*
NCG	verbena	revetlla	*open-air dance*	arracada	*earring*
NCG	pañuelo	mocador	*handkerchief*	concert	*concert*
NCG	zumbido	brunzit	*buzz*	altaveu	*speaker*
NCG	vivienda	habitatge	*home*	desenllaç	*outcome*
NCG	oscurecer	enfosquir	*darken*	degenerar	*degenerate*
NCG	pesadilla	malson	*nightmare*	teulat	*roof*
NCG	subir	pujar	*go up*	matar	*to kill*
NCG	azote	flagell	*scourge*	arxiduc	*archduke*
NCG	chica	noia	*girl*	font	*source*
NCG	entero	sencer	*whole*	difunt	*deceased*
NCG	cierre	tancament	*closing*	trimestre	*quarter*
NCG	cobijo	aixopluc	*shelter*	aturador	*breakdown*
NCG	empezar	començar	*to start*	conèixer	*to meet*
NCG	enferma	malalta	*sick*	vegetal	*vegetable*
NCG	búsqueda	cerca	*search*	lliga	*league*
NCG	averiguar	esbrinar	*find out*	suprimir	*to remove*
NCG	encuentro	trobada	*meeting*	portada	*cover*

NCG	carcajada	riallada	*laugh*	intuició	*intuition*
NCG	envoltura	embolcall	*envelope*	assistent	*wizard*
NCG	detener	aturar	*to stop*	actuar	*to act*
NCG	ahorrar	estalviar	*to save*	reproduir	*to play*
NCG	eco	ressò	*echo*	exili	*exile*
NCG	bebé	nadó	*baby*	anís	*anise*
NCG	chico	noi	*boy*	cau	*nest*
NCG	borde	vora	*edge*	vers	*verse*
NCG	fresa	maduixa	*strawberry*	altitut	*altitude*
NCG	buzón	bústia	*mailbox*	còpula	*copulation*
NCG	ahorro	estalvi	*saving*	estímul	*stimulus*
NCG	charla	xerrada	*chat*	baldufa	*spinning top*
NCG	charco	toll	*puddle*	duel	*duel*
NCG	barrer	escombrar	*sweep*	anticipar	*to anticipate*
NCG	cadera	maluc	*hip*	faldó	*shirttail*
NCG	espejo	mirall	*mirror*	dimoni	*devil*
NCG	flotar	surar	*to float*	untar	*spread*
NCG	huella	empremta	*footprint*	revolada	*stirred*
NCG	cuerno	banya	*horn*	sogra	*mother-in-law*
NCG	azotea	terrat	*rooftop*	ritual	*ritual*
NCG	cuerpo	cos	*body*	cel	*sky*
NCG	azúcar	sucre	*sugar*	patró	*pattern*
NCG	entrega	lliurament	*delivery*	pessimisme	*pessimism*
NCG	asiento	seient	*seat*	gravat	*engraved*
NCG	cuartel	caserna	*headquarters*	quimera	*chimera*
NCG	calidad	qualitat	*quality*	muntanya	*mountain*
NCG	desnudo	nu	*naked*	pi	*Pine tree*
NCG	agujero	forat	*hole*	hotel	*hotel*
NCG	espesor	gruix	*thickness*	claste	*worship*

NCG	bandeja	safata	*tray*	careta	*mask*
NCG	colchón	matalàs	*mattress*	balancí	*rocking chair*
NCG	ajedrez	escacs	*chess*	sumari	*summary*
NCG	entregar	lliurar	*to deliver*	asseure	*sit down*
NCG	cuchillo	ganivet	*knife*	dipòsit	*deposit*
NCG	ensalada	amanida	*salad*	àlgebra	*algebra*
NCG	apellido	cognom	*surname*	lleure	*recreation*
NCG	desnudez	nuesa	*nakedness*	pugna	*rivalry*
NCG	desprecio	menyspreu	*contempt*	homenatge	*tribute*
NCG	disfrutar	gaudir	*to enjoy*	bullir	*to boil*
NCG	discípulo	deixeble	*disciple*	llinatge	*lineage*
NCG	cordillera	serralada	*mountain range*	corrupció	*corruption*
NCG	carpintero	fuster	*carpenter*	bigoti	*moustache*
NCG	escaparate	aparador	*showcase*	convidat	*guest*
NCG	baloncesto	bàsquet	*basketball*	altiplà	*plateau*
NCG	cortar	tallar	*to cut*	marxar	*to leave*
NCG	esconder	amagar	*to hide*	sofrir	*suffer*
NCG	alquilar	llogar	*to rent*	voltar	*come back*
NCG	elegir	triar	*to choose*	curar	*to cure*
NCG	niñez	infantesa	*childhood*	identitat	*identity*
NCG	llanto	plor	*crying*	suro	*cork*
NCG	mezclar	barrejar	*to mix*	emportar	*take away*
NCG	neblina	boirina	*fog*	llacuna	*lagoon*
NCG	merienda	berenar	*snack*	alumini	*aluminum*
NCG	meter	ficar	*to put*	alçar	*rise up*
NCG	luto	dol	*mourning*	arc	*bow*
NCG	jugo	suc	*juice*	veí	*neighbor*
NCG	noche	nit	*night*	nom	*name*
NCG	mujer	dona	*woman*	obra	*work*

NCG	miedo	por	*fear*	pau	*peace*
NCG	muslo	cuixa	*thigh*	reixa	*grate*
NCG	musgo	molsa	*moss*	núvia	*girlfriend*
NCG	mezcla	barreja	*mixture*	reforma	*reform*
NCG	jueves	dijous	*Thursday*	reflex	*reflex*
NCG	locura	bogeria	*madness*	palanca	*lever*
NCG	marfil	ivori	*ivory*	illot	*islet*
NCG	manzana	poma	*apple*	fila	*row*
NCG	mendigo	captaire	*beggar*	portador	*bearer*
NCG	mariposa	papallona	*butterfly*	normativa	*regulations*
NCG	medición	mesurament	*measurement*	absentisme	*absenteeism*
NCG	llenar	omplir	*fill*	emprar	*use*
NCG	lavar	rentar	*to wash*	vèncer	*to win*
NCG	limpiar	netejar	*clean*	adormir	*to sleep*
